# Supplementary material for: Understanding barriers to implementing referral procedures in the rural and semi-urban district hospitals in Tanzania: Experiences of healthcare providers working in maternity units
Source: PLoS One. 2021 Aug 26;16(8):e0255475. doi: 10.1371/journal.pone.0255475 (PMC8389460; doi:10.1371/journal.pone.0255475)
Supplement: S1 Data — (ZIP) [file pone.0255475.s001.zip › Supporting documents/Dataset-Referral System Implementation.doc]

**DOMAIN 15: REFFERAL SYSTEM**

<Internals\\MK1000_K_01> - § 1 reference coded [9.33% Coverage]

Reference 1 - 9.33% Coverage

IQ: Thank you very much, now let us go to the next area, I am sorry I have many questions but the aim is to understand better. Now let us look on referral system. Please tell us how the referral system works in your setting

RR: Worry not, just asks as much as you can, after all you are asking interesting things. Back to your question, as a health centre we receive patients from all dispensaries under our catchment. We treat those cases we can manage here and or those we find that they are beyond our capacity we send them to the district hospital.

IP: How many dispensaries are under your catchment?

RR: Currently we have six dispensaries from 10 villages.

IQ: How do you send patients to the district hospital, in this case I am referring to the pregnant women who needed emergency help, do you have ambulance?

RR: We used to have an ambulance, but since it broke two years ago and taken by the district for repair it was never brought back. So what we do know, when we need to refer we call for help from the district hospital by phone call. Sometimes we get and sometimes we do not get, when we miss we tell the patient and relatives to try their own means to reach there.

IP: Is there any feedback system in your referral system? Like do you provide feedback to the dispensaries on patients referred to you and receive feedback from the district hospital for the patient you referred to them?

RR: There is no feedback… we do not give feedback and I have not received any feedback from the district hospital. But, I think it is a good thing. Thank you for bringing it I am writing it here we will start to do that to our dispensaries, please also suggest it when you talk to the district hospital management.

IP: Has the referral system changed with time? That is to say maybe sometimes the referral was this way and now you do this and that which were not there before.

RR: No since I joined the health sector the referral system is like that; from dispensary to health centre, from health centre to district hospital, from district hospital to regional hospital and then from there to Muhimbili or Bugando Mwanza. What has changed is the availability of ambulances. Before it used to be that every health centre had an ambulance but now some have and some which is the majority do not have.

<Internals\\MK1000_K_02> - § 1 reference coded [11.05% Coverage]

Reference 1 - 11.05% Coverage

IP: What is the role of the AMO in the referral process?

RR: The AMO is the final authorizer that this patient is taken to higher facility and at our level is Kasulu district hospital. His role is to make an examination of the patient and write a referral for the patient.

IQ: What are the challenges facing the referral system?

RR: The main challenge we face now is that we do not have an ambulance, we used to have one and it got an accident, when it was taken to be fixed it was never brought back. So when we want to refer a patient we have to call Kasulu and request for an ambulance. Sometimes we get and sometimes we fail.

IP: What do you do when you miss an ambulance?

RR: We tell the patient and relatives to find an alternative transport.

IQ: Is there a feedback mechanism in the system of referral?

RR: I have never seen it, once we refer we are done with that business unless is a patient you have interest with you can call a friend there and ask the ABC of that patient.

IP: Has the referral system changed with time?

RR: Since I joined the health sector the system has remained the same.

<Internals\\MK1000_W_02> - § 3 references coded [18.87% Coverage]

Reference 1 - 3.51% Coverage

IQ: Now, can you please describe the referral system .Tell us how is the referral system in this facility?

RR: Referral system?

IP: Yes

RR: Sometimes referral System works, due to shortage of power/electricity problem when maintenance is done.

IR: ok,

RR: Here we have eight sets only, and if there is no power the equipments will remain unwashed/unsterilized, as there some times all sets can be used in a single day.

IR: ok

RR: Therefore if we have used all the equipments, and there is no electricity we must refer the patient to other hospital. Kabange hospital.

IR: ok,

IR: ok

RR: Yes,

IR: Ok.

IP: How do they treat them/receive them? Kabange hospital?

RR: They are attended well.

IP: Can you please tell me on the challenges of the referral system and how they affect performance of caesarean section by assistant medical officers?

Reference 2 - 9.76% Coverage

RR: challenges are sometimes there, as there sometimes refusing to receive patient from the facility.

IP: In case of such situation what do you do?

RR: You will find a way/other means on how to help that patient; you can’t just leave them hanging.

IR: ok,

RR: Yah,

IP: Have you ever referred a patient there and refused to attend he/them?

RR: Yes, it happened...

IP: So how was it? Was she sent back to this facility??

RR: Exactly,we brought her back.

IR: Ok,

IP: Ok, ok, and how is the feedback mechanism? How do you get feedback after being referred there?

RR: feedback mechanism?

IP: EE, feedback

RR: The hospital where we refers our patients, asks us why do you bring us patients? Why don’t you treat them at your facility and bring them here? Sometimes you try to explain to them that is due to shortage of equipments……

IR: Yes,

RR: So they refuse to receive them, as results the patient/s are sent back our facility.

IP: What about the refereed patients? How do you get their results /feedback? How do you communicate between your hospital and the others

RR: The patients usually go with the escorter together with the referral letter. After reaching the referred hosp the escorter will receive the feedback regarding the patient whether he /she will be treated there or have to come back if not received by the doctor the referred hospital. However there is no any explanations from the doctor of the referred hosp all explanations will be given by the escorter.

IR: oooh

RR: yes

IR: OK ok

IP: Who usually escort the patient?

RR: Nurse

IP: Ok is a Nurse, do you also receive patients who referred by the other hospitals?

RR: Yes, We do receive

IR: From which hospitals then?

RR: Most of the patients who are attended here are the once referred

IR: ooh

RR: Eee, yes

IP: Suppose the referred patients come here and find that some of the devices are not available, what do you do?

RR: We usually go on doing resuscitation until other preparations are ready then we attend the patient.

IR: Ok

RR: Yes

IP: Have this system of referring patients being changing from the 15 years you have been at work? Or is it the same? Give us your views.

RR: Since Kabanga Hosp has refused to receive the referred patients, we never take our patients there anymore

IP: So what do you do then?

RR: We just attend them here at the district hospital

Reference 3 - 5.59% Coverage

IP: You have told us about the referred cases from here to Kabanga

RR: yes

IQ: What about the referred cases from the other health facilities to this facility?

RR: A re you talking of the referrals from health centers to our hospitals?

IR: yes

RR: For the referred cases from the other low level health facilities to ours I think no problems. The challenge is that sometimes with the referred cases the diagnosis is usually not the real one coz after we do see the patients we find them with other problems /diagnosis not the once stated by the original health facility.

IR: Not the real one?

RR: yes

IP: What do you think about the referrals? Do they make your working environment difficult when delayed?

RR: YES

IP: oooh

RR: when delayed some of the patients die.

IP: What is the other problem with referrals from the low level health facilities to the upper levels like yours?

RR: Lower level facilities can stay with patients for quite a long time and when they come to bring them you find that it is already late

Interruption (“some people are talking”)

RR: So some patients will arrive here already with the problem of rapture of uterus.

IQ: You have been working here for a very long time! What do you think the assistant medical officers are…..?

Interruption (the doo was opened…the interviewee went to close the door)

<Internals\\MK1000_W_08> - § 2 references coded [10.15% Coverage]

Reference 1 - 9.01% Coverage

IQ; Thank you, the referral system….how does the referral system in your settings facilitate or hamper a woman in need of referral after getting problems that cannot be handled at your facility during caesarean sections?

RR; hhhhhmmm….that the mother is pregnant and after delivery she requires a referral?

IR; eehhhh

RR; in general during the period that I have worked here, I have never had a pregnant woman requiring a referral. The complications that we see are fistulas. But that has never been a problem because we have…..vvf surgery nearby in a mission hospital about 8 kilometers from our facility. We take our patients there for the management. But for the case of other major complications, we have never had any of those. But in general there is a problem with the referral system in two main ways….for patients who are not staff members we face problems whenever there is no fuel…..this is the major obstacle. You may have a patient in need and whom you are sure that they will benefit more if they are referred to Bugando or Muhimbili or any other place…..but you may find out that the family is not capable of handling the costs and at the same time the budget we have does not allow to cater for the fuel expenses.

Otherwise…..the bigger problem is with the staffs….the referral system oppresses the staffs for example whenever a staff requires referral for a particular care, at the district hospitals we cannot refer them directly and the care that they need is not available at the regional hospital and they will not get it and I have communicated with someone at the regional hospital and they confirmed the absence of such services and otherwise maybe they should just go to pay for the transport costs for it is a must for the referrals to pass through the regional hospitals, therefore sometimes as a doctor I may know that we do not provide a certain service and it is a must for them to go through the regional hospitals for the go ahead in getting their benefits. So this is a system that puts obstacles and limitations in the availability of services in time most especially to the staff members.

IP; thank you very much…is there a system of getting feedback whenever you have referred a patient to another center?

RR; When you return, may you come see me so that we may know what the problem was and how it was managed. Only a few of the patients return to the district after their treatment but some of them do come. And the other problem is that even after treatment and discharge, they are given a discharge summary that is not self-explanatory. You will find that someone is just given prescription for medications and there is no a summary explaining what was found and the management given so you end in the dark without knowing the progress of the patient.

IP; you have mentioned some of the places where you refer your patients too…. There is Bugando or Muhimbili…. What kind of transport is being used to take patients to such facilities. I think in the case of going to Muhimbili, if you leave today you will arrive tomorrow when travelling by bus. What kind of transport is being used,…. Do they go by plane… or by car?

RR; …..the means of transport….. first of all our referral center is Bugando in this zone. But there are times when you know that this particular service is not available at Bugando, maybe I am forced to refer them directly and sending them to Bugando will be unnecessary circle that will delay their service; and if the patient is financially capable to pay for their own transport fee, you let them know that you would like to refer them directly to Muhimbili because referring them according to the policies there will be no any services rather they will end up referring you to some other place. So the transport that we use most of the time is the ambulance so if the patients have been referred to Bugando, you fill in the fuel and the patients are taken.

IP; How many hours does it take….from rough estimations from here to Bugando?

RR; it takes eight hours to 12, it depends if there are no any obstacles on the way. In a few times the patients are being taken by plane but the transport costs are usually upon the patients so if they are financially stable and they can pay for the transport fee by plane we support them by providing an ambulance to take them to Kigoma airport and then from there they go by plane.

Reference 2 - 1.14% Coverage

IP; how about in the case staffs….. is the situation the same….. (silence….noises outside)…. Are they being taken by ambulances as well or by plane?

RR; we also take them by ambulance….if we are taking them to Kigoma but….since I came I have not had an ill servant who required transportation by plane due to their condition. But all that I came cross we transported them by ambulance …..and even if they required transportation by plane, we will have to take them by the ambulance up to Kigoma because we do not have direct flights within the district.

<Internals\\MK1000_W_09 (1)> - § 2 references coded [18.48% Coverage]

Reference 1 - 11.31% Coverage

IQ: Can you tell us how the referral system affects delivery trough caesarean section done by assistant medical officers. I mean the current referral system that is present, how does it work first of all?

RQ: We are talking about a pregnant woman or to all in general?

IR: Especially to pregnant woman

RR: That referral has….

IR: I mean how are those referrals affect delivery through operation which are done by AMO?

RR: until they refer a person?

IR:Mmmm

RR: For example… those who are at the health centres or?

IR: Mmmmhu…..

RR: for instance those who are at the health centres don’t do operations.

IR: Mmmmhu

RR: they don’t have theatres

IR:Mmmmmh

RR: If they get a woman who needs operation they refer her to the district hospital, where there are doctors, there are AMOs, who will discuss themselves and operate her.

IR: Mmmmmhuu

2IP: where do your referrals come from? Where do you get patients from?

RR: health centres in the villages and dispensaries.

2IP: how many are they? Don’t you know its total?

RR: They are many

IR: Okay

IP: what challenges that you get in this kind of the referral system?

2IP: Don’t you receive referrals from health centres?

RR:Mmmmmh

2IP: Maybe a case is sent here, and it becomes quite difficult to you, how is the referral system?.....

RR: For example, they can refer a mother due to prolonged labour, but when she arrives here she gives birth normally. So you might find that they refer patients without fundamental reasons. For example, these prime gravid, they delay giving birth, so they shouldn’t be worried to stay with that patient, until they see a bad indication that is when they should refer her.

IP: Okay. I want to know how do those referrals enable or affect delivery through operation. Either are they delayed or? I want to know how referrals affect giving birth through operations which is done by AMO?

RR: If a woman

IR: Mmmmh….

RR: there is no any effect. But now a woman can be referred from the village and fails to get transport, and delays to get here. When she gets here then she is in bad situation. When she gets here they will operate on her.

IR:Aaaa… okay

IP: Can you add on a little bit about challenges that you face due to the current referral system, because you have told us that a mother can be delayed, are there any other challenges?

RR: Can you explain vividly.

IR: I mean that that referral system where you receive people, from villages so that they deliver their babies, are there any other challenges you face?

RR: A mother can get referral, but comes without any letter. You fail to know where to start ( …noise…..a person is coming in and is welcome…). A person is referred from nay be a health centre, and was told to go to Kasulu, and there is no letter showing why was she referred. So when she arrives here you start afresh

Reference 2 - 7.17% Coverage

IR: Eeee, they are like those that you have mentioned, do you have others?

IP: what are the roles of assistant medical doctors in those referrals?

RR: If a doctor receives a woman who has been referred here from a health centre, he checks if he can operate her. If he cannot then he asks for another help

IP: Aaaaa okay

IP: How about feedback after giving referral? You sometimes refer people, isn’t that so?

RR: There was a time that they were giving referrals

IP: Eee, now you have referred a person to somewhere, or you have received a referral, how is the system of feedback?

RR: If we received a person without a letter, then the person who has received her communicate to the facility that has referred her, telling that person that next time he shouldn’t do so, that he shouldn’t refer a person without information, so that we can get where to start.

IR: Okay

IP: How about feedback on the situation of the patient?

RR: Maybe if she will be discharge safely, we communicate with them telling how is the patient

IP: and when you refer a person to somewhere else?

RR: You write a referral letter explaining why you have referred her. Then later on you communicate. If the reason to refer is not satisfactory. You are told that is not the referral, if they think that thing can be done here.

IR: Aaaah okay.

IP: Okay. Which changes have happened in the referral system, which you can say previously in certain years things were like this, and now it is like this.

RR: I cannot know about previous years

IR: Okay, how about years that you have been here?

IR: Aaaah okay. So you don’t know about any changes?

RR: I know nothing; I have been here for a very short time

IQ: what is your opinion, on how we can improve our referral system?

RR: Infrastructure should be improved

<Internals\\MK2000_W_01> - § 6 references coded [12.92% Coverage]

Reference 1 - 1.40% Coverage

IP: is there a time, (eeh) when then numbers were maybe too high or too low?

RR: For instance last year, I can say, (mmmh) it was three but this year, (mhmmm) they have increased to ten (ten…) yes

IP: So what do you think is the problem there?

RR: The problem if you ask them they say its……it is just late referral, (uuh huh) the patient is brought when they are in a critical conditional and most of them are not from delays from our central hospitals, for instance,(mmmh) most are from Nanyumbu council.

IR: Uh huh

RR: for example..or those from Masasi Dc, you will find that they bring us, (eeeh) till the time our doctors come to handle that work it is already too late, eeeh (okay) and as of right now a death is not recorded as where the patient was from but at the place of death, (mmh) and that is why it seems like we have lots of deaths.

Reference 2 - 1.29% Coverage

IQ: okay, now let’s talk about the referral system, referral of….of patients, (mmmh) I would like to know firstly how the referral system here is

RR: the referral system, (yes) for instance a case has proved too challenging here, (yes) for us here we use Ndanda hospital as the referral hospital yes, for example if a situation couldn’t be dealt with here (yes) the doctor writes referral to, and we follow the government’s policy of free service for pregnant women.

IR: yes

RR: that is we give her a free referral to there, (yes) and children under five years too, (yes) but for others the procedure, we produce a car, and a driver the remaining things are upon him / her, he / she will provide the money for fuel, (the patient?) the patient will be responsible, provide twenty litres.

IR: okay

Reference 3 - 6.63% Coverage

RR: Because the trip to Ndanda and back is twenty litres, so he / she will provide twenty litres, the driver will take him / her, (yes) and the car will return.

IP: uh huh, and what if he / she can’t provide those twenty litres?

RR: If he / she can’t provide those twenty litres, the hospital incurs the cost (mhmmm) we provide it.

IQ: an….nd now that is the process of patients from here to other hospitals, what about those patients from other hospitals coming here? Do you have any such cases?

RR: yes there are

IR: What hospitals are they from?

RR: there are those from centres in Masasi district council, (mmmh) there are those from Nanyumbu, (okay) most come from those areas.

IQ: aaa..nd.. In what way can you say this system affects “….” the functioning of or the responsibilities of performing caesarean operations done by Assistant medical officerss?

RR: the challenges are quite large there, (mmmh) because…..(mmh) first the budget we get, the government calculates with respect to our population, (okay) so when we get additional patients it becomes somewhat troublesome.

IR: yes

RR: the meaning now, they come it’s not like they come with their tools and what not, no, they use ours, (mmmh) sometimes they come here and we don’t have those utilities or sometimes and there is only one surgical bed left

IR: okay

RR: so the patient comes in and the doctor performs the caesarean operation, (mmmh) while at the same time there is another patient from Mangaka also requiring a caesarean operation (mmmmh) and there is only one bed it becomes somewhat of a challenge

IR: mmh

RR: So working tools, (mmmmh) man power too……man power I can say is not too much of a problem if only there was another bed, another doctor would have been called, (mmhmmm) and he / she would have come to assist, (yes) but the tools, that is the challenge we have

IR: yes

RR: The other patient now maybe needs to be taken straight to Ndanda, (mmmh) it becomes hard, because we here don’t have that much fuel, the fuel available lasts only until Mkomaindo, (mmmmh) so we end up having to dip into our pockets to transport that patient with our personal money, (mmmh) transport them to Ndanda (uh huuuh) yes

IQ: aa…..and what things do you think enable this whole referral process

RQ: enablers?

IR: yes, the ones that enable the responsibilities of those Assistant medical officers

RQ: do you mean the referrals we receive or the ones out from here?

IR: both, the ones we…..you receive and those that go out

RR: for those that we receive we have started a system, they have to call beforehand…..that is before they send a patient here, (mhmmm) they have to call first, we are bringing a patient with the following condition and they will receive a response and before he / she leaves there they will get a response whether bring the patient (mhmmm) or maybe don’t take him / her to Ndanda because we have a certain problem here.

IR: okay

RR: that is the system that has been introduced, (okay) from here to where we refer them to, there is no problem there

IQ: and what is the feedback system after referral or the information transfer.

RR: aaah feedback for the patient, for a patient who has beed referred? (Yes) honestly, there is no feedback, feedback (mmhh) I think is provided during the morning clinical meeting.

IR: mmhmm

RR: the nurse on duty will provide the whole report if there was a referred patient or something, but feedback for development of the patient honestly we don’t receive that, (mmmh) after we send them there that’s it, (uh huuh)

IP: and have you had any way in which you have been trying to follow up on that feedback.

RR: no there isn’t.

IP: and is this how it has been since you started work or has it ever been different?

RR: no, that’s how it’s always been

IR: that’s the way it is?

RR: mmmmh

IP: what about you providing feedback to where you received patients from

RR: no, we don’t provide it

IR: you don’t

RR: yes, (okay)

IQ: and now….that system “….” in your opinion how can that feedback system be improved

Reference 4 - 0.71% Coverage

IQ: now what is the role of an assistant Medical Officers or in the whole referral process of, “.....” especially for those women?

RR: this assistant has a responsibility of, (mmh) only writing those…filling that referral form, that is his / her only role

IP: okay, and after he / she fills who goes to that other place?

RR: the nurse on duty, (okay) she is the one who looks for a way to go with the patient to where they are going, (okay) yes

Reference 5 - 0.62% Coverage

IP: eeh now suppose a patient comes and there is no blood to be found

RR: it becomes his / her responsibility to look for blood.

IP: what if they can’t find any one?

RR: …(laughs)…. If he / she can’t find anyone, we take him / her to Ndanda, (uh huh… okay) yes, that is if a patient requires blood and there is no blood and no one was found, (yes) we refer him / her to Ndanda

Reference 6 - 2.27% Coverage

I2Q: okay, lets please talk about those referrals that you receive, (yes) how is the system of the referrals that you receive like, there are these that you send out, you say they go to Ndanda, (yes) what about the ones you receive from below, how are they like?

RR: these that come in from below, if for example there is an issue……a case occurred and there is an issue they couldn’t solve, (mmmmh) they call and give a heads up that, there ‘s a patient required to come to Mkomaindo and maybe she has a delivery problem and we have failed or maybe she has failed to deliver so that’s how the information is like so the important thing is that provision of information, and a nurse leaves from here and goes for that patient yes “……” because they have no cars in the centers.

I2P: what system do you use, is it radio calls or just normal phones?

RR: no, just normal phones

I2Q: Okay, (eeh) and on the issue of purchase of like things for theatre, (yes) which are required in caesarean operations, these AMOs “…….” Are they involved in the process?

RR: no they aren’t involved, (mmmh) because they normally have a representative we just take…. It’s not like….everybody can participate NO! Because for purchases there is a special committee that’s been formed which represents purchases, we’ve tried to pick from “……” every department a single person.

I2R: mmmh

RR: yes, he / she will be representing

<Internals\\MK2000_W_05> - § 4 references coded [13.50% Coverage]

Reference 1 - 7.71% Coverage

IQ: Let’s now talk about the referral system how does the referral system works for someone who needs the service of delivery through operation works? Maybe if I bring a patient from the low level facility what happens when she reaches here and even someone who has suffered from complications here?

RR : when they receive a patient, take care of her, and failed to handle her, they decide to refer the patient to district hospital, but in many cases we have our ambulance, they call us, they we send the car to pick up the patient. Like our colleagues who stay far away, for instance the nearby district who don’t have a district hospital, they decide to bring the patient directly to the district hospital. When she is brought at the district hospital she is sent to labour ward. Those nurses will examine her and if they see that they can handle her, she needs doctor’s assistance, then the nurse on duty or overall nurse is told so, and calls the doctor to come and examine that woman. The doctor is the one who will decide if that woman will undergo an operation or any other treatment

IP: When it happens that there is a big complication and needs to be referred to higher hospital, what is the procedure?

RR: the procedure is that we write a letter for instance we don’t have certain equipment or something, we need to refer her, we write a letter and take that patient to Ndanda referral hospital, in many cases we refer them to Ndanda, that we have failed to handle her because of one reason or the other.

IP: Specifically what is the reason in many cases?

RR: the main reason is that you can have a breakdown of certain equipment, maybe sterilizer or we don’t have blood in our blood bank and the patient needs blood, so we are supposed to rush her to Ndanda where they have blood in many cases

IP: Do you have a system of getting feedback for those you refer to far hospital and do you provide feedback to dispensaries which refer patients to you? That your patient that you brought here now is like this and that?

RR: To be sincere we do that rarely, and even them to send feedback is rarely

IP: How do see the system of feedback?

RR: It’s a good one. Because for instance you have failed to handle a patient and decide to refer her to Ndanda, you would like to know how your colleagues handled that case so that it will help you next time

IP: The way your referral system works, what should be improved so that it works well?

RR: Basically what enables us is the presence of ambulance …to be honest it helps us a lot

IP: How many ambulances do you have?

RR: We have on ambulance

IP: How many facilities does it serve?

RR: now the district council …because we have two councils; district council and town council. We have five facilities, including dispensaries and hospitals, but our colleagues, they have more than forty dispensaries. In many cases they request our ambulance if they have a patient, maybe sometime we don’t have fuel they put fuel and the cars goes to pick that patient and bring her to district hospital.

IP: Eee when you talk about town council, district council, where is their hospital?

RR: district council don’t have a hospital

IP: do they have ambulance?

RR: they don’t have an ambulance.

IP: But they serve dispensaries?

RR: They have about forty dispensaries

Reference 2 - 1.57% Coverage

IP: How do they serve them, if they don’t have a hospital and ambulance, or maybe you help them serving those dispensaries now?

RR: In case of emergency patients for instance mothers who failed to give birth at their dispensaries, they have a car which is not an ambulance, rarely do they use that car, but often they ask an ambulance from us. That they have a patient and they need an ambulance, we send the ambulance to them. However we face fuel challenge, sometime we tell them that here is the car but don’t have fuel, they say we will contribute fuel. We send an ambulance to them to pick that patient and bring her to our hospital, and we continue with service as usual

Reference 3 - 1.82% Coverage

IP: To what extent is the referral system affect delivery through operations which are done by assistant medical officers?

RR: Its effects are that sometime referral cases are delayed, a patient can stay for a long time in the village, so until she is brought here, she will be delayed, and start even to have complications and bearing in mind that our district hospitals don’t have many equipment for operation. So when a person comes with complication that causes problem a lot in operation. For instance our hospital depends more on table, so when you find that a patient have many complications and there is no oxygen gas, and there is AMBU machine. So you find that you are in a big trouble, you are doing an operation and the same time praying to God that I shouldn’t get DIT here

Reference 4 - 2.40% Coverage

IP: You were telling me challenges which are faced by referral system, to what extent has this system changed according to time?

RR: previously every health facility had a car and it was easy for patients around that area to be its is not in the health centre instead it is in the district. Because there are other activities in the district and they don’t have cars so they decide to take those cars from health centres and send them to other activities. Now we have two two districts. Town district and which has started last year or two years ago. So that is a challenge because the district council doesn’t have a hospital, and don’t have a car, so they rely on the town council. In their health centres our collegues in the district council, they have three health centres. I don’t think they have any car in all those health centres. They rely on this ambulance from another district. If you have bad relationship, they will tell you have your council, go there. In providing services Tanzanians don’t have those things.

<Internals\\MK2000_W_07> - § 3 references coded [15.09% Coverage]

Reference 1 - 7.04% Coverage

IP: Have you ever had a patient who needed emergency Caesar and there were no instruments? What do you do to help her?

RR: Mother is referred to a nearby mission hospital in Ndanda called Saint Benedict

IQ: we are approaching the end section four is about referral can u tell us how referral system operates

RR: Referral system here maybe after u realize patient needs operation but there are no instruments is referred to Ndanda and is seen by ward doctor for instance here in labour ward there are three doctors, so after they are seen by doctor, I as ward in charge have role to tell medical officer in charge who prepares transport to send patient, and Is escorted by nurse and has a filled referral form

IQ: and what about referrals that you receive how are the protocol?

RR: Referrals that we receive are from a certain hospital called Ndungama, patient is brought by a nurse with referral letter which is handled to us on arrival, we then trace doctor who comes and review patient and if there are decisions for Caesar then patient goes for Caesar

IP: So are your referrals from Nanyumbu?

RR: It’s a district and health Centre but they have started performing caesarean section, u may find one doctor and one anaesthetist so most patients are referred here.

Reference 2 - 4.05% Coverage

IQ: That is what you go through what about the ones you receive

RR: There are challenges u may find delay of patients, mother may have uterine rupture, obstructed labour lasting for two days once they realize they can’t manage that’s when they refer so late referrals from one Centre to another and that’s why most ruptured uterus occur here

IQ: Do you have system in which u follow up on patients you referred to see what has been done on her?

RR: We have no system of follow up, once we send that’s it

IQ: U wash hands off

RR: Yes we wash hands off to be honest

IQ: What can you say about referral system, you have been here for long time, and is there any change in this referral system?

RR: I have not seen any changes

Reference 3 - 3.99% Coverage

IQ2: Thank you, my last question I would like to quote you, you said you get referrals and sometimes you send referrals to Ndanda I want know what reasons are for transfer to Ndanda

RR: it’s not all the times, its few times maybe two or three patients, most it’s because there are no stitches so u can’t do operation without stitches because as u do operation u have to stitch to prevent bleeding there was a patient brought in for Caesar from Mpanda but due to lack of stitches we had to refer her to Ndanda so u may find shortage of instruments

IQ2: So problem is instruments?

RR: Its issue of instruments only sending patient to Ndanda

IQ2: How far is it to Ndanda?

RR: It’s like 35 minutes from here to Ndanda

<Internals\\MK3000_C_02> - § 1 reference coded [2.13% Coverage]

Reference 1 - 2.13% Coverage

IR; meaning that the training starts at school then to clinical practice, at the hospital, so from the hospital environment and infrastructures and everything available that may assist and facilitate training in performing C/S, do you think that the infrastructures and equipment are available correctly?

RR; the infrastructures are good as well as the availability of equipment

IQ; what about other requirements such as medications, blood?

RR; Ahaa, there is a problem with that sometimes there is no blood, someone maybe referred for blood transfusion maybe go to KCMC, due to lack of blood, same applies to medications sometime in the past we had problem with blood due to lack of reagents for checking if the blood has no infection, this was a very big problem, sometimes we could not perform an operation due to lack of blood

<Internals\\MK3000_M_01> - § 1 reference coded [15.61% Coverage]

Reference 1 - 15.61% Coverage

IQ; How is the referral system to the person who needs it enables cesarean operation? In which ways is the referral system to a person who needs to deliver through caesarean section works?

RR; when they are referred from the district to here?

IR; First of….

RR; we work well because nearly all councils have ambulance for referring patients. Also in those councils there are competent doctors who can do cesarean sections. So it’s not that we don’t get patients who are referred here. We get them. So from dispensary to a health centre, to a district hospital. We have vehicles for referrals cases. Now as a region we have strategy of enabling health centres do operations, so that there won’t be a long process of referring from a dispensary to district or referral hospital. So the referral system is good.

IP; and how about the ones that you receive is there no…?

RR; we have them, we receive them from this city council because they don’t have a district hospital, they also look at the location they are. For instance we have a health centre which is in Pongwe, on the way to Dar es Salaam which does the operation. So those who are in that area go there for operations. While those who are in here in town, come directly to Bombo for operations

IP; how about feedback after referral?

RR; We have feedback. For instance in every week we have reports of mothers who have delivered, and those who delivered through operation, and where do they come from. So we send the report to the district so that it helps them in planning, so that they know where majority of women come from e.g. from a certain dispensary so that they upgrade that dispensary to a health centre with a theatre so that mothers won’t be traveling for a long distance. So we give them feedback.

IP: What things which enable you. You have said that there are cars for referrals, what else enable that referral system to work as you have explained?

RR; others are going to those clinics which they attend antenatal clinic, assessment is done to them in those clinics to determine if a person is at risk? For instance a person who had cesarean section previously, and she becomes pregnant again, so she has s previous scar, so you will know definitely that person can fail to deliver in a normal way; so it’s better you start preparations to refer her before labour pain starts, and informing her that she should stay near a hospital where you know that she be served well. So we have a system.

IP; Okay, Thank you. Which obstacles do you face in the referral system we have talked about?

RR; challenges are there because you will inform people about something but others don’t follow up, because they see themselves as adult so she doesn’t follow. For example we got one case in Pagan district. You know there is a river in the other side of Pangani. Unfortunately on the other side one of our health centres doesn’t have a theatre, but now they are in the process of building a theatre. So one of the pregnant women was told that she had a scar, she had an operation previously. She was told that when delivery time approaches she should go early to the other side of the river so that she we be served immediately. She didn’t listen to that. She went to a festival and labour pains started; it took a long time to bring her to the hospital. So when she was brought here her uterus has ruptured, and died. So we advice them but sometimes people don’t listen; there is usage of herbal medicines; that you have been told that your labour is difficult; but when she goes back home other will tell him that so and so used herbal medicines from a certain man and helped her; so even you go and use them they will help you. And she goes and uses them. You find that she uses that medicine and gets labour pain quickly and uterus ruptures and brings disaster. So there is a problem with regards to education. The education needs to be sustainable to people so that they know the importance of be informed by a health provider. It’s good to go quickly to get operation services near the place that you live; rather than staying at home and use incorrect medicines.

IP; okay ….. How can we improve referral system?

RR; what we do is to improve for instance for us referral starts f at all levels. There is a community level, health centre, district hospital up to referral. So at the community level that means we have to continue educating people on the importance of going early to clinics so that her situation is assessed and directed the proper place to deliver her baby. On the other side of supervisors of health services at the regional level should make sure that we have emergency theatres in our health centres, so as to simplify the referral system; Even in our district hospitals and should have employees who can do them [ operations] because you can have theatre rooms, but you don’t have workers to do those operations; so at the same time and we should have referral vehicles in case of complications at district hospital then she should be referred to regional hospital?

<Internals\\MK3000_W_01> - § 3 references coded [8.42% Coverage]

Reference 1 - 7.24% Coverage

IQ: to what extent is the referral system is to someone who need cesarean section service works?

RR: we have distributed cars and ambulances

IR: mmhu

RR: We have distributed four ambulances

IR: Yes

RR: one of the ambulances is in Mkata and another one is in Chogo

IR: mmhu

RR: there were refugees at one time

IR: Yes

RR: we said that there should be an ambulance there, and we have sent another ambulance to Kabuku

IR: Yes

RR: and there another ambulance which remains at the hospital

IR: Yes

RR: so that it can go around these areas

IR: ok

RR: We have agreed with the council that in case of obstetric emergencies they should sent a car and fetch them

IR: ok

RR: and there is no cost sharing

IR: ok

RR: there is no cost sharing, fortunately we have not faced any problem in those obstetric emergencies. We have not referred them to regional hospital, we did them here.

IR: mmhu

RR: unless we find out that a person has …….. For example this week we have a patient admitted with cardiac lesion [*cardiac lesion*] he is suffering from faint attacks….

IR: Yes

RR: and is pregnant, so we must work hard before labour pains we have to send him to regional hospital, so we will give transport to see a gynaecologist ….

IR: Yes

RR: there is a physician, he will take a good care of her, and will make a decision, and even the anaesthetist will know how to monitor her

IR: ok

RR: so that they help that mother to deliver

IR: ok

RR: so we refer those patients with that nature to higher level hospital

Ryes

RR: but currently there is large amount of referrals from lower facilities to district hospital

IR: ok

RR: But in a few days that will decrease, because Mkata dispensary will be finished and it will be operating

IR: Yes

RR: because a ward has been built already to receive those women. Currently the ward is in use; however the theatre has not started operating

IR: Ahaa, Thank you so much

IP: how is the feedback system after referral?

RR: Ohooo…..that is the area which has small weakness here, however when they discharge her they don’t put a note there.

IR: ok

RR: you should write to the person who has referred that patient

IR: ok

RR: letting him know that you thank him. To thank him for taking that decision first, because that is more than ability

IR: Yes

RR: you thank him that we have received the patient, and we have served her very well. We have done, 1, 2, 3, and now we are returning her to you, you should make a follow up on this and that

IR: Yes

RR: That is an area with weakness.

IR: ok

RR: eee, which is a weakness. I can see that majority of them are discharged with a discharge form and not referral forms for feedback

IP: Apart from that…. …..weakness, what other challenges that you face in the referral system?

RR: sometimes fuel is a crisis

IR:Mmhu

RR: fuel is a real big problem; you get a problem and find that fuel is a problem

IR: Yes

RR: what do you tell a patient for instance those who are nearby area, to send her to Kabuku? Or a relative hires a car, so that is a challenge

IR: Ok, right, we thank you so much

IP: To what extent is the whole referral system in providing delivery services through operation?

RR: Effects to this

IR: Yes

RR: if she has referral, and the patient continues to remain there searching for another transport, psychologically he will be affected. No one likes his relative to die

IR: It’s true

RR: eee, in one way or the other, the patient that you are serving longs to die, even if she has no obstetric case

IR: ok

RR: You find that you have wasted your energy and time and at the end of the day the patient dies, no one likes that

IR: ok

RR: what if it happens that you have failed to refer her because there is no fuel or the car is not working psychologically that hurts

IR: Yes

RR: doesn’t want that, it hurts her

IR: ok

RR: even the facility performance l

IR: yes

RR: you want the perfomance of your facility to be good

IR: Yes

RR: that it is supposed to provide quality care. So you will find people saying that if you go there and you are in critical condition, you can’t be referred, until you look for another car

IR: OK

RR: if there is a nearby health facility, and they are able to go there, then they will go there rather than coming to your facility

IR: ok

RR: Lastly, people will be going to other facility for delivery, not at your facility because there are services but the problem of transport will stuck her

IR: ok, thank you very much

IP: In general, can you mention things which will enable this referral system to work well?

RR: Ahaa, first of all is the availability of those cars and running cost budget should be there, because those cars need services after certain kilometers

IR: Yes

RR: They need services

IR: Yes

RR: ability to buy spear parts should be there

IR: ok

RR: fuel should be there throughout

IR: ok

Reference 2 - 0.84% Coverage

RR: A motorcycle was brought here for referring patients, however it is not working well, and it doesn’t turn right, where can you find roads that turn left only? None.

IR: None

RR: We have dismantled those motorcycles and sent them to remote areas, because we need them to bring report to us before seventh of every month, so that we enter them in to DHS2. So we have given those motorcycles to peripheral areas and they use them to bring reports and not to refer patients.

Iron, its right, thanks you very much. Let’s talk about the infrastructure and equipment

RR: Mmhuu

Reference 3 - 0.34% Coverage

IQ: Do you have cases here whereby AMOs has been employed directly from college without….

RR: No, there is none

IR: Ok, there is none

RR: We have none. All of them have upgraded

IR: Ok, we are so thankful for your participation

<Internals\\MK3000_W_03> - § 4 references coded [8.87% Coverage]

Reference 1 - 2.43% Coverage

IQ: Let us talk about the referral system here how is it?

RR: Mmh?

IR: I mean referring a patient to referral hospital or a patient has been referred here from a dispensary

RR: Majority of them are referred here from health centres

IR: Mmhu

RR: They come with letters…..

IR: Mmhu

RR: The one who comes from for instance Mkata and Kabuku come with their referral letters from health centres

RR: mmhu

RR: But the ones who comes from margin villages from small dispensaries

RR: mmhu

RR: they don’t come with letters

IR: mmhu

RR: Eee you will find that she is only escorted by a nurse in that car

RR: mmhu

RR: because they come in private only

Reference 2 - 2.27% Coverage

IR: mmhu

RR: eee, they come without letters

IP: Now to what extent is that system affect assistant medical doctors work?

RR:…. The assistant medical doctor is supposed to take that patient

IR: mmhu

RR: and starts asking about her history

IR: mmhu

RR: the history of that mother history

IR:Mmhu

RR: That how did it start from when she was home up to when she went to dispensary?

IR:Mmhu

RR: and from that dispendary up to the time they decide to bring her here?

IR:Mmhu

RR: Eee, they are supposed to take her history for a long time

IR: Mmhu

RR: so that he understand the problem of that woman

Reference 3 - 3.28% Coverage

IR: Mmhu

RR: Mmhu

IP: How many referrals can you receive in a day?

RR: mmh?

IR: of pregnant mothers who come to deliver through cesarean section?

RR: …….(thinking……)

IP: or in a month?

RR: In a day you receive three, another day two, and another one you receive one of patient ….

IR: Mmhu

RR: They don’t have a specific number

IR: Ahaa…..

IP: what challenges do you face in receive those referrals?

RR: Challenges that we face you can find that when that mother is operated on the stomach…

IR:Mmhu

RR: You find that her stomach has strange smell

IR: Mmhu

RR: Whe you get the mother like that

IR: Mmhu

RR: First if you ask her before operating on her

IR: Mmhu

RR: She will tell that labor pain has started two days ago when I was at the dispensary

IR:	Mmhu

RR: When she is brought up here, we will find smell inside her stomach even if we start giving her antibiotics.

Reference 4 - 0.89% Coverage

IQ: using your twelve years experience is that system the same or has changed sometime?

RR:…..with regard to referral?

IR:eee referral

RR :…..(he is thinking…..) telling the truth I have not seen changes

IR: You have not seen changes?

<Internals\\MK3000_W_07> - § 1 reference coded [2.98% Coverage]

Reference 1 - 2.98% Coverage

IQ: Where do you refer your patients to?

RR: Bombo

IP: What challenges do you get in the referral system?

RR: There are a lot of challenges …..You mean when you give a patient referral for obstetric care?

IR: Yeah referral for obstetric care

RR: there are few challenges regarding referral for obstetric care, I thought other referrals because you might find out the ambulance have got no fuel so we have to make patients contribute in order to be taken to the referred hospital.

IP: Those are the challenges you get while referring, what about the challenges you get when you get referral?

RR: They are the same challenges since the ambulances are at the health centers standby to bring patients but sometimes you might find the car does not have enough fuel to go back to the health center.

IR: ok

<Internals\\MK3000_W_08> - § 3 references coded [12.01% Coverage]

Reference 1 - 10.21% Coverage

IQ; yeah thanks perhaps let’s look on the fifth part that is concerning how they refer and how is the referral system for those who need caesarean section service work?

RQ; referral to the much bigger hospitals?

IR; yeah referral to much bigger hospitals or you receiving from the small facilities the one enter and the ones left what are the procedures

RR; for us most of the time if you take these maternity case very few occasion we refer especially the one that the individual is obese and has pressure that she need physician and gynaecologist to do team work we usually refer but for here its much worse most of the patient brought here with the condition that even if I refer he/she wont stays longer its better we do it here so you do for risk though there is other way the patient might not be having money to go to other facility just been brought by the motorcycle she borrow the motorcycle owner or he bail her thing so that the patient get services most of the time this maternity case we don’t refer because of the patient condition that they come here with bad financial status.

IP; but here some that are brought here that you see they are in condition that even when you refer they won’t reach you think....?

RR; the big issue is that most of the villages are very far coming from the village to dispensary is quite far and majority of them depend on available traditional birth attendant they are some of the birth attendant that don’t have their place so they come direct they say let us go to the hospital, big hospital so you find they are coming without passing to the dispensary some of them just went to the antenatal clinic just once or haven’t gone even twice they are some of the facilities that the RCH of this hospital has to go so during the time when there was no fuel they didn’t go that mean there was no service delivered so if you find has attended once or twice she come when the labour has already stated maybe you find she is in second stage you find such things happening

IQ; ok ok and perhaps what are the facilitators in the all referral system meaning when we look at the barriers and those things that made the referral to run smooth?

RR; aahh the main issue is fuel because when you refer the patient the car must have fuel so that you can refer the patient now there s no fuel so maybe the patient is well financially maybe we refer the patient to Bombo Tanga the distance for go and return needs about 80 litres so 80 litres for here you find the hospital can provide 40 litres and relative they have to contribute 40 litres so if you find relative don’t have other are telling you do anything because even where you I am going I don’t have money to eat and the patient can’t go alone by herself so those are the challenges we are facing

IQ; yeah and probably, when you refer is there any procedure for the feedback|?

RR; aaa usually most of the time when we give the referral we aren’t given the feedback it is just once or twice trice we refer cases to Muhimbili that when we had a feedback of the patient he returned here with the letter and they explain his what but when we refer to Tanga we aren’t given feedback its only Muhimbili and KCMC......KCMC them it just few times not all the time that is when we have feedback the patience you referred had this problem and will continue attending clinic so if she get emergence she will be coming here and we be using what medication and you find the tell the patient when you arrive there send her to the one who brought them here so we at least we know what is going on

IQ; ok and how is the referral system affecting assistant medical officer in delivering service of cesarean section?

RQ; hhhh I don’t no perhaps you should repeat a question

IR; yeah is just that ok this all process or the all procedure for these referral you might in one way or the other it interfere our other schedule now we specifically look for that these assistant medical officers.......especially in the caesarean section that how does this all referral procedure affects if you look at both sides it might be negative or positive that caesarean section they are doing?

RR; aah honestly there heheh…. I real don’t know that how I should explain of that but most of the time they specifically can’t say this patient has to go certain place without the medical officer arriving there if there is the thing that happen during the night and need emergence that very night medical officer must be given information so that he comes if it is a transport officer has to come at that time the referral planning start but individual person as individual it is difficult so if it is an emergence then the medical officer is informed first and he then he/she come with the whole team then the referral system

IP; here is where he gives and what about when he receive maybe?

RR; honestly in receiving we don’t have any rules or what because we know when the patient is brought here remember most of them the rent a car you may find Noah arrive here brought one of the thing or some time motorcycle or what so you find they come in that way majority are in that way I told you that they are in the bad condition so you find at the same time some other time she come without a letter without any explanation so maybe relative may give the information you might find some have forget their clinic card at home so they come as they are so here you just have to take fresh information do urgent investigations if it is emergence and need caesarean section she is sent to theatre for caesarean section but majority they don’t have that what but for most of those who attend clinic you find their cards are written which hospital will she give birth at end.....

Reference 2 - 0.46% Coverage

IQ; yeah thanks and probably in brief maybe when we mean responsibilities of assistant medical of officer in the referral system what are those?

RR; hhh honestly I can’t recall much for those heheh perhaps hospital secretary can answer that question nicely

Reference 3 - 1.35% Coverage

IP; and perhaps if we look at their responsibility like you have explain in this referral that those responsibility have been changing time after time or the role that he/she is suppose to apply during the referral have been in changing ?

RR; meaning if changes

RR; previously most of the time they were referring themselves directly

IP; Them directly?

RR; Enheeeee and even now their some circumstances that himself/herself who writes the letter but the medical officer in charge must come and be around during that time of the problem for that maternity case he must be there and transport officer if it’s the case that need..But as I have said early for most of the maternity cases the way they arrive here meaning you can’t refer as she might push

<Internals\\MK4000_C_01> - § 1 reference coded [5.57% Coverage]

Reference 1 - 5.57% Coverage

I2Q: From you understanding is there anything specific that was changed?

RR: No Maybe because I was involved in teaching I am not sure.

I2Q: As I understand that the current training is under NACTE, when do you think that the new program will start?

RR: We will start when it will be passed

I2Q: As I understand another challenge is there is no carrier ladder unlike doctors and that they seem to run parallel what about other challenges that AMO are currently facing how are we sure that they won’t keep on facing them when it becomes Bachelor degree.

RR; It may not be my field but some of these challenges are why it has been difficult to pass it.

12R; mm

RR; Ee now it is this other doctor’s turn, who is he?

12R; mm

RR; Of course it has reached a point where the medical council has declared that there is no a four years degree. The curriculum that has been made in place of AMO says degree will be for four years so the meeting will have a big challenge with regard to the curriculum.

12R; mm

RR; so how are the relevant authorities going to solve this because there will have two different degree programmes offering the same thing. Yet this discipline is different from others, in the computer courses you can create a course and call it whatever name and people will learn but it is different here in the medical field as we have a systematic way of operating, a doctor doesn’t just work like that.

<Internals\\MK4000_K_01 (2)> - § 3 references coded [9.42% Coverage]

Reference 1 - 5.28% Coverage

IR: thank you, let’s go to the fifth section, about the referral system.

RR: okay,

IQ: can you explain to us how the referral system affects the c/section procedures performed by AMOs, for instance the referral patients that you receive, how do they affect the c/section procedures performed by AMOs?

RQ: affect in which sense? To Patients or workers? As I mentioned before our theatre has all the necessary equipments.

IQ: what about the referrals that you give?

RR: they are very few, we only refer when we really have a problem at the theatre, and I have never referred an expecting mother.

IQ: what about the ones you send?

RR: most of them are due to other medical causes.

IQ: Yes, how does the referral system affect your efficiency in performing c/section?

RR: the efficiency is affected when we get late referrals and receive patients in worse conditions, because the ambulance has to bring them from about 60 km away, and we use the facility’s funds for fuel.

IQ: what about the patients you refer to other facilities?

RR: the patients that we refer are taken by our ambulance to Ifakara hospital.

IQ: can you explain to us how the referral system works?

RR: there is no any system in particular; if the patient has to be referred they leave by the ambulance immediately.

IQ: what challenges do you face?

RR: nowadays we don’t have a lot of challenges due to CH, we finish almost everything right here.

IQ: is that a referral system?

RR: we don’t refer most of our maternal cases nowadays.

IQ: what about the ones you receive?

RR: we receive most of our referral patients from Ngeta, Masangati. There are no ambulances in peripheral facilities so patients are brought by Hiace, Noah, motor cycle or Bajaji, which cause a lot of delays and patients arrive here in worse conditions and some with complications already, so they give us a hard time in managing them.

Reference 2 - 1.55% Coverage

IQ: and how does the referral system affect the c/section procedure performed by AMOs? What is your opinion on this?

RR: we receive only few referral cases nowadays, unless there patient already has VVF then there is nothing we can do we just refer the patient to Ifakara.

IQ: are there any feedback arrangements?

RR: we don’t have any feedback arrangements, but most of the time when we refer patients they come back at our facility after being treated there.

IQ: are any timely changes to the referral system?

RR: there are no any timely changes.

Reference 3 - 2.59% Coverage

IQ: how effective is the c/section performed by AMOs?

RR: that’s why I told you we do the procedures at our facility without referring patients, that means we are good at our job despite the hard working environment, we try our best to help our people, we are so proud of that.

IQ: how is the ability of AMOs to prevent poor delivery outcomes? And how do other cadres contribute in this?

RR: we meet every Friday and remind ourselves some aspects of our work, we also have a computer which gives us access to learning materials for example if you want to read about PPH or Drum ears you just find it on the computer, and when we meet on Fridays at 3pm we discuss administrative and clinical issues, the nurses also get time to ask the doctors how to do things or doses of drugs, so don’t be surprised when a nurse attendant performs like a midwife, it’s because of their experience and on job teachings that we do ourselves.

<Internals\\MK4000_K_03> - § 3 references coded [25.20% Coverage]

Reference 1 - 10.45% Coverage

IR: Let’s talk about the fourth part, which is on referral system.

IQ: Can you tell us how the referral system works and how it affects the effectiveness of surgeries done by doctors?

RQ: What?

IR: Referrals

RQ: The ones we refer?

IR: I mean the ones you get and the ones you refer.

RR: Okay, we rarely refer patients, and I don’t clearly understand the referrals we get.

IQ: I mean the referrals you get from the village dispensaries, are there any effects?

RR: Yes there are effects because some women come with ruptured membranes and they have to be operated. Most of them delay coming to the hospital so at the end of the day complications may arise and we have to save the mother and leave the child to die, while if they came earlier we could save both of them.

IQ: And the few referrals you do, how do they affect surgeries?

RR: We have few referrals like since I worked here in 2010, we have referred only two patients, and they pay normally. The main problem is transport, but if there is transport, then they are taken as normal.

IQ:

RQ: The referral?

IR: Yes.

RR: It becomes difficult to explain because we rarely refer patients from our facility, most patients are treated here and discharged home.

IQ: What about the referrals you get from the villages?

RR: It is not easy, we have to inform the mother about her condition, why it has happened and that if she did not delay coming to the hospital she wouldn’t have had any problems. It also depends on how far she came from, plus the transport issue because the roads are bad, and some patients come from very remote areas

Reference 2 - 14.11% Coverage

IQ: The role of doctors in how these referral systems work, how does it affect delivery through C/S?

RQ: Their responsibilities?

IR: Yes.

RQ: In referring patients?

IR: I mean how the whole referral system and the role of doctors, do they affect delivery through C/S in any way?

RR: I do not think there are any effects.

IQ: Are there any feedbacks you get or give?

RQ: After referring patients?

IR: Yes.

RR: Yes we get feedback from the escort nurse and sometimes we call the patient and know what is going on. Our doctors communicate with doctors at St Francis hospital so they tell us how the patient is doing.

IQ: Do you also give feedback for patients you receive from villages?

RQ: Giving feedback from our center?
IR: Yes maybe do you give them feedback?

RQ: Giving feedback is from our center right?

IR: You are the ones receiving them from the villages.

RQ: Okay, giving feedback to their villages?
IR: Yes.
RR: Yes we give feedback because we also go to those villages giving medical services and explaining how they can overcome the health problems they get. They also come for clinic so they understand because we explain to them the problems, risk factors, what can be treated.

IQ: Are there any changes in the referral system as time goes by?
RQ: Changes?
IR: I mean the referral system, the way it goes, are there changes as time passes by?

RR: I do not see any changes.

IQ: How is it?
RR: It is good, but they are few referrals like in 5 years we have referred only two patients.

IQ: And when you receive patients from the villages, what do they come with like any documents that show they are referred, or how is their referral system?

RR: They have written documents, the dispensary doctors write the reasons for referral.
IR: Alright.

RR: Yes and when the patients get here we check them and know the problem or sometimes they come with a diagnosis so we manage them. Some referred patients deliver normally and some by operation.

IQ: So they write a referral letter?

RR: Yes they do.

IR: Okay.

IQ: And what do you do here when you refer patients to Ifakara?

RR: We have referral forms.

IQ: You do?

RR: Yes.

Reference 3 - 0.64% Coverage

So you refer patients if the anaesthetist or AMO are absent?

RR: Yes, because there are very few.

<Internals\\MK4000_K_04> - § 3 references coded [14.27% Coverage]

Reference 1 - 6.29% Coverage

IR: Thank you very much, we are now approaching to area 6 let us discuss a little about the system of referral

IQ: please can you explain to me on how the referral system affects caesarean section done by medical assistants in the sense of referrals that are received and services that you offer.

RR: for sure referrals we received in the past it was difficult, some arrive here while they have ruptured the membrane because long travelling distance, others are coming seventy kilometers like for ones comes from Msagati, their roads are in bad shape especially during rainy season, but we have already discussed about this and we have built a maternity waiting room here so those who may think that they are supposed to give birth at here can get emergency care they may come here, so at least we don't get so often referrals who are in critical state or we hardly transfer patients to Ifakara, it can even take us at least six months without referring to Ifakara.

IQ: What are the Assistant Medical Officer’s responsibilities in the referral system?

RR: His responsibilities is to see if he can manage those cases here or to take the patient to hospital, so those are things are checked during antenatal clinic or labor.

IQ: And how do you receive patients from seventy kilometers away from here and referrer them, are there any system of giving and receiving feedback?

RR: there are special written form but let say there is little possibilities of getting feedback, there are special form found here written feedback from receiving facility, but it is very difficult for a person to return to you feedback, so there are special forms for feedback but rarely do we send them back

IQ: And how about you, do you give feedback to the lower level?

RR: Sometimes patient don't come with this form or a patient comes straight from home so it becomes difficult to give feedback to lower levels.

IQ: what are the challenges in this referral system?

RR: the challenges are long distance, other times transport is the problem, we can say like that, transport is not durable, we can say that cars themselves are broken and are not in good condition.

IQ: how many cars do you have for referral?

RR: we have one ambulance

IQ: is it working all the whole time?

RR: it is working though it has small problems.

Reference 2 - 2.32% Coverage

IQ: for your own perception what do you think on the mechanisms that we can use to improve this referral system?

RR: i think for this referral system in the previous time all ambulances had radio call, but for now those radio call aren’t present, but these days we have our mobile phones, maybe we have to improve because every station have mobile phones and there are some people IHI established this program but they didn’t work properly.

IQ: explain a little about this system of radio call and what do you think how this referral system will be improved?

RR: for example someone can be in station A at Msagati calls for transport because he has a patient who is in critical condition, so we send the car immediately to bring patient or I make a call informing them about the status of the patient, so when they reach there, they get the right service.

Reference 3 - 5.66% Coverage

IR: Thank you so much probably there is additional question from my assistant researcher that I have probably didn't ask.

IR: Thank you, I think the important issues you have already said, maybe issues concerning promotion, you have said earlier that there were no good procedures and then you entered to OPRAS system, I would like to know after the uses of OPRAS system in promotion, do you think is it a solution to a problem of been promoted in time and how do you talk about the system?

RR: That is not exactly the solution because you might find that someone is promoted but the salary remains the same it is also a problem , and if you went to the office that deals with the salary issues, they will tell you it is still pending, and it can stay in pending even in five or six months it is a problem and to be promoted it also needs follow-up, and but if they could do like other offices we heard about them only after three month someone is promoted, so this system it is not the solution but the situation at least is not bad as it was before.

IQ: And probably there is issue of referral, you have said that it is not most of the time that you get the feedback, though your forms are design in a way that you should get the feedback, the one responsible to return the feedback is it the patient or the receiving hospital?

RR: it is that receiving hospital, I am thinking that if the patient is from here and they didn't clarified who is to bring the form, but just a piece of it as you can see it needs a feedback, so the problem is how we return it, should you ask the patient to return it or how would you return it, it is also a problem

IR: Thank you so much for your cooperation and you time it is our hope that we will get the chance to give you the feedback regarding with this research as we spoke earlier, if you will have any questions do not hesitate to contact professor Masada through the numbers which are in the form that I have approved to you, the information that you have provided to us there are so important to facilitate this research, Thank you so much.

<Internals\\MK4000_M_01> - § 2 references coded [7.81% Coverage]

Reference 1 - 6.34% Coverage

1Q: Thanks, Now let’s go to referral section how the referral section work in caesareans section?

RR: For a caesarean section persona this is done according to the assessment that is done by doctors it see the patient need this services. They assess then selves in their station and if see a person needs referral they refer him or her. Example from dispensaries to health centers it they can’t handle they go to the next level. But this is done according to the assessment of that problem.

1Q: What’s about the system of feedback after referral?

RQ: There is that system but is not but recent there was a meeting discussing about the system of referral on how to get feedback. So the system starts from the underground stations to the regional level through RMO and see how the doctor fill the forms.

1Q: Are there facilities in this system of referral? And what are facilities that help you to work as you explained?

RR: First these are standardized tools that are used to give feedback example ambulances have been used to bring mothers to the next level of service.

IR: Mm

RR: But there are those things you explained earlier quarterly maternal auditing review. Meeting which among other it discusses the issue of referral so as to improve it more.

1Q: Ok, Thanks how do this referral system affect caesarean section services?

RQ: Affect in appositive or negative side?

IR: In any side

RR: First let me start with positive that this referral system help mother to deliver safely well and even if she got complications it needs scissor maybe she have obstructed labor so this is rushed to the next level of services and deliver a child safely then mother become happy.

IR: Mm

RR: And the child comes safely .other challenges of this referral system is distance where some area is too remote. Example if you find one has engaged in obstructed labor to get in an ambulance and in some area ambulance is so far which delay to reach the destination point. Another challenge is that the feedback system is not good. So next time motivation is needed. So we don’t have a good system to ensure that the one goes to the next level of service there is a feedback from where he or she comes

Reference 2 - 1.47% Coverage

1Q: Maybe Doctor. Tell us what are the responsibilities of assistance medical officer in referral to refer somebody.

RR: Mm

1Q: Yes AMO had what responsibility to referral system?

RR: Assistance medical gives referral as other doctors if they assess the patient according to his or her condition may refer him or her to next level of service as other doctors.

1Q: How those responsibilities change according to time?

RR: I don’t know if the referral system has changed over time?

IR: Ok thanks.

RR: Mm.

<Internals\\MK4000_W_01> - § 2 references coded [10.51% Coverage]

Reference 1 - 6.46% Coverage

IQ: How does the referral system work to the particular person?

RR: In this centre we have ambulance we also caesarean ambulance, mobile phone of which in case of any emergency they are connected. They are also having connection with referral hospitals. For instance there is an emergency and it’s beyond their capacity we transfer him/her to referral hospital our ambulance has got a nurse who escort him and they.

IQ: Do they communicate with you before they bring from the health centre?

RR: Yes, they do communicate direct with the referral hospital.

IQ; What if in referral hospital fail. What do they do?

RR: When referral fails it to attend the patient they will take him to Muhimbili.

IQ: Is there any feedback after the patient has been taken to the hospital?

RR: Of what happened?

IR: Yes.

RR: Personally I have not seen but I can report with DHIS and he has been referral what is the outcome, I can see in the report.

IQ: How do you see the availability of facilities in referral system what facilitators in referral system.

RR: In the referral system there is ambulance, I believe the council provide everything for instance fail technical support they do provide everything to the patients.

IQ: Any restrictions.

RR; There’s no restriction by then we only face problem when the car has been taken to maintenance and the money used is taken is of other charges money brought late so the tendered fail to repair because of not been paid. When waiting for the car to be repaired health facilities has an alternative the system which collect money for this service of this can take a car and bring him

IQ: How does the referral system affect the work performance of the assistance medical office?

RR: Yes, certainly if death occurs in his health facility it will demoralize him in that health centre there must be martenal auditing and other things. If this occurs in his health facilities the health facility basin in the council its real affect.

Reference 2 - 4.05% Coverage

IQ: Basing on that referral system does the responsibilities of assistance doctor changed over time?

RR: It has changed as before it has been established it has a lot of problems. Such that it could bring patients for more than firth to sixth times within a day. They are the caesarean cases the reference system has improved a bit because after been established it has reduced the caesarean case.

IQ: As you can see on the issue availability of infructure and equipment, how can it be improved?

RR: In our council, we have to make arrangements of that the equipment and other supplies they are available for instance in basket fund about 67percent is for medicine and equipment.

IQ: How does AMO’s participated and ensures availability of infrastructure and equipment in caesarean section?

RR: In procurement section, practically we have district pharmacist through Integrated Logistic System-(ILS), transfer city they are to request for their supplies and this is done by in charge of the station if it happen that the in charge is not the AMO the caesarean performer is not likely to be involved. When she can recognize the needs besides we have something known as buffer stock that we can buy so as to top up but I don’t think if there is direct involvement.

<Internals\\MK4000_W_02> - § 4 references coded [8.04% Coverage]

Reference 1 - 3.24% Coverage

IQ; may you explain to me how does the referral system affects the tasks carried out by AMOs in performing caesarean section?

RQ; meaning?

IR; for example, do the referrals that you receive affect you performance at work?

RQ; referrals from other health centers?

IR; Eee, or dispensaries.

RR; they do not affect me, but it’s just that when a patient comes in a critical condition I don’t have an ICU, it really affects me that I fail to help the patient, if it is carrying out the delivery and continue the management in an ICU which would be lifesaving but I cannot do that, what are you going to do if you face a complication, will you refer them, they have been referred and you end up referring them again, you see, it is really troublesome to the patients, but these are challenges that are out of my control but with time the system may improve and we may have the ICU and other wards

IQ; so how does the referral system operate at your facility?

RR; Ahaa, we accept the referred patients just as usual and we work on that system and if you can manage the case then you work on it but if it is out of your capacity then you refer them, but at some time you may end not seen the patient because you are staying far from the hospital and a nurse after seeing that patient may conclude that nothing much can be done at our facility and they refer them without been seen by the doctor, so that happens a lot and you end up missing on the opportunities for learning but in some occasions the patient may have been referred for an ambulance and you know that your facility has no ambulance so they end referring them quickly before development of any complications due to delays, so you don’t get an opportunity to see the patient for the experience so you will hear about that case later on and that it was referred due to so and so reasons, it was seen by the pm doctor or the one on the night shift and you will be left out.

Reference 2 - 0.32% Coverage

IQ; so what is the responsibility of an AMO in referring patients?

RR; we are responsible in attending to the patients if we can, but if you cannot you document what was out of your capacity\

Reference 3 - 2.22% Coverage

IQ; and how is the system of getting you feedback, like from the small centers that are referring the patients?

RR; honestly you have just reminded us, most of the time they don’t do that, they will only make a call because I am not the in charge, so they inform the in charge over the phone on asking about the progress of their patient and that is when we get them feedback but that rarely happens

IQ; and do you get feedback upon referring a patient?

RR; we will get feedback if the patient was mismanaged and let’s say the patient died that is when we will get feedback, and the feedback is usually condemning that we are useless and completely irresponsible, you see, but if the management was successful nobody will inform you

IQ; and the referrals that you see, have they changed over time or are they still the same?

RQ; Mh?

IR; has the referral system changed? If you compare when you started working, in the mid and currently has it been the same?

RR; it has been the same, the referral system has not changed, maybe it’s just that in the past we used to document a lot more content, we used to document on a paper all the explanations about the patient but currently the ministry has limited us on that, it should be a brief history, brief examination and provisional diagnosis and your management in summary

Reference 4 - 2.25% Coverage

IQ; so what are the challenges in the referral system?

RR; the challenge that we are facing is that at times we do not follow protocols, when you don’t have infrastructures such an ambulance you just inform them even without a nurse and sometimes without documentation because when you document you will have to escort the patient and sometimes everybody will be escaping from that, so when they see that you have come with a Bajaj they ask the Bajaj to wait and they will take a brief history of the mother, and inform them that the doctor is currently not available so just go to another facility, so at the end of the day we are not doing them justice and they are sick at that moment, the regulations is that the information of the patient should be well documented and an escort should take them to the other facility because anything can happen on the way, who will help them, so that is still a big challenge the facilities are not enough, today the ambulance maybe working, on the next day it may not, so that is how it is and a nurse has to escort the patient, so when you look at it, they need escorting and coming back and unfortunately the patient has no money, so at times they go alone without an escort because the patient will have to pay transport fee for the nurse as well although that is how the system is supposed to be.

<Internals\\MK4000_W_03> - § 3 references coded [17.11% Coverage]

Reference 1 - 5.25% Coverage

IQ;	ok, thank you. We are entering another section, we are just finishing here we don’t have long discussion. We are look at referral system. Please tell us how the referral system works here?

RQ;	referral?

IQ;	ee for the one who needs to deliver through operation

RR;	mmh, here referrals occurs, how do they happen? For instance after examining the patient and see that she has a problem, we call doctor on duty who comes and check on her. Also we see if there is an MD or AMO, if they are on call or at home so that he can come and help us. Sometime it happens that MDs are not around, it happens so many times, and sometimes AMOs are not there, may be they are on holidays. What we do is calling a doctor because others are not there, they come and prepare referral

IQ;	ok. How about those referrals that you receive?

RR;	I receive few of them, in many cases they deliver in a normal way, so we receive them and some of them are not for scissors

IQ;	how do you handle them? How is does the system works there? From when the patient is referred and moves from there to here where you receive her?

RR;	we receive them. I have received one mother, she didn’t have a problem with referral, and she was referred here because she stayed for a long time without falling pregnant. She stayed for ten years without falling pregnant, since her first pregnancy. So we examined her and found that she has no problem. We checked her HB, and everything was okay. Fortunately she delivered normally

Reference 2 - 8.04% Coverage

came because she stayed for a long time without falling pregnant that was the criterion to refer here

RR;	ee that one was escorted by a nurse and fortunately that nurse was from here

So she was here until that woman delivered. Because they were worried that she would get PPH and where she stays was far away and there are no cars there. So they brought her here so if a problem arises we can send her to St Francis because we have a car

IQ;	and what would have happened if the nurse was from that area?

RR; because we know where they come from, we communicate with those nurses because we have their numbers

IQ;	how do you communicate?

RR;	by telephones

IQ;	so you have….

RR;	ee communication is there

IQ;	anhaa ok. Thank you.

RR;	mm

IQ;	what challenges are there in referral system that you have explained?

RR;	another challenges that we face is that another day when you send a referral to St Francis, they have said that if you take a patient there and you don’t find an adult person maybe you find that there are students and interns they don’t like to receive the referral, so you have to stay with your patient. But we tell them the reasons to refer that patient for instance the doctor is not around etc. Another challenge is car sometimes it troubles us

IQ;	troubles

RR;	there is a problem

IQ;	isn’t it there or what is the problem?

RR;	sometime is not working

IR;	amah

RR;	mm

IR;	ok

RR;	challenges are there

IQ;	any challenge maybe? Anywhere even to patient?

RR; to patient is a big challenge because hat is a private hospital, everything is money, something which is different from here, we do operation free of charge, and you leave when you are done. I think when they go there they pay. Even when you tell a patient that you are sending her to St Francis they don’t receive that wee, they are hurt

IR;	mm

RR;	they are thinking about money mmhh

IQ; are there any changes that have emerged in this referral system, although you have said that you have been here for a short time, but at least you know

RR; I see that there are no many referrals because we have all doctors

IQ;	and that helps to reduce or increase?

RR;	I mean they are decreasing, even us we don’t receive many referrals

IQ;	how about you referring patients?

RR;	we don’t refer many

Reference 3 - 3.83% Coverage

I2Q;	right, can you explain more when you said you refer many cases

RR;	mm

I2Q;	where there is no doctor

RR;	mm

I2Q;	and sometimes that causes you to face challenges when you refer those cases to higher level hospital

RR;	ee

I2Q;	do you think there are other reasons, apart from absence of doctors. Is there any other reason that causes you to refer a patient to St. Francis?

RR;	many cases

I2Q;	especially mothers

RQ;	mothers?

I2R;	mm

RR;	in many cases it’s like that if doctor who does the operation is not around

I2Q;	mm

RR; a few times that I have faced are like that mmh

I2R;	mm doctor

RR;	ee if the doctor is not around is the problem

I2Q; in which environment do you find the doctor is not around?

RR;	e.g. when he is on holiday

I2R;	mm

RR;	if some of MDs have gone to seminars, then it becomes a problem because we don’t have many doctors. What I know four of them do operation. There are two MDs and two AMOs who do operation

I2R;	mm

RR;	so that is a challenge, one of them can be on holiday, another one in a seminar, so that is a problem

I2R;	mm

RR;	mm

<Internals\\MK4000_W_04> - § 8 references coded [14.94% Coverage]

Reference 1 - 2.98% Coverage

IQ;	Thanks, I am moving to another section on referral system

RR;	mmh

IQ;	How does the referral system works for a person who needs delivery through operations? First of all do you have the system?

RQ;	of referral?

IR;	mm

RR;	its there

IP;	mmh, how is that? Tell us how that work does

RR;	referral is at all levels, from the community, dispensary, health centre to hospital

IR;	mmh

RR;	in the community there are workers who are taught for nine months. We have Ifakara centre organization which taught these workers for nine months on how to handle mothers, how to refer them and to determine dangerous indications, so they were taught many things. So they make follow up in that community and when a mother falls pregnant and goes to clinic and given her ABCD. These community health workers make a follow up to those mothers in the community, so the referrals start from the community. They insist to mothers to go to dispensary, if she won’t be able to deliver in that dispensary they refer her to a health centre.

IR;	mm

RR;	mm and there is a special form that they fill in. when she arrives at the health centre, if she has dangerous indications, or has scissor indications and that health centre does operations then they operate on her there, if they don’t they refer him to the hospital

IR;	mm

RR;	mm

IQ;	Ahaa gives referrals in those levels

RR;	in charge

IP;	in charge?

RR;	in charge of the dispensary, in charge of health centre

IQ;	mm, and what if in those all levels ….

RR;	mmh

IR;	as a district, how do you know that there are patients who are referred?

RR;	as a district every month we get referrals from the facilities

IR;	mm

RQ;	Yes, do you know MTUHA?

IR;	mm

RR; Yes, this MTUHA has different books for referrals, there are there, so if they have referred a patient they will fill in there in that book

IR;	mm

RR;	when a report comes every month you know that a certain centre has referred a patient

IP;	mmh so in the district level, you will know the number of mothers who are referred in different levels

Reference 2 - 1.38% Coverage

RR;	yah

IQ;	When we come to the hospital level, can you know that for instance a patient was referred from a health centre to district hospital?

RR;	mmh

IQ;	like this Kibaoni right?

RR;	mm

IQ;	how do those people in the health centre know what was the outcome of that mother who they have referred? Is there such system in the district?

RR;	[Coughing…] there is a referral form and there is a feedback, however, there is negligence of some kind because feedback forms are not filled in

IR;	mm

RR;	mm now we want to insist on that so that those who receive patients fill in and return it to the respective facility

IR;	mm

RR;	but those forms for referring and returning her feedback are there

IR;	mm

RR;	mm

IQ;	so what does that form explain? How is that form? Have you seen that form?

RR;	that form explains mother’s situation and why is she referred, and in that feedback from tells what was done to that mother and what the outcome was

Reference 3 - 2.46% Coverage

IQ;	right, apart from that form is there any other form like a forum or something else?

RQ: mmh forum for?

IQ;	I mean, apart from that form is there any feedback system?

RQ: feedback?

IR;	Yes

RR;	yah its there because every Thursday we have maternal auditing meeting at St Francis, and here RCH CO goes there, they say that how many referrals they have received, and what were the reasons, if deaths has occurred they discuss them there, what was the reasons and where has that death occurred, they discuss and reach consensus. When they come back from that meeting they give us feedback

IR;	mm

RR;	yah.

IQ;	right, can you tell what are there to enable this system of referrals? What facilitate this referral system to work well?

RR;	mmmh, what facilitate, first of all is that we have ambulances which help to refer those women

IR;	mm

RR;	we have motorcycles which are made special for mothers, and when they refer for instance, if they come from the dispensary, the ambulance goes there to pick up that mother and a nurse escorts the patient up to the respective facility

IR;	mmh

RR;	mm.

IQ;	Apart from that system of taking mothers by ambulance to a referral facility?

RR;	mmh

IQ;	you have said that there are special ambulance and motocycles

RR;	mmh

IQ;	for transporting mothers

RR;	mm

IQ;	is there another thing which facilitate that referral system to work well?

RR;	mmmmh another thing is telephone- communication, that before a patient moves from facility A the one who will receive her is called that a certain mother with certain symptoms is coming , so that they prepare a doctor and other to be ready for that

IR;	mm

RR;	so that facilitates too

IR;	ok.

RR;	ee.

Reference 4 - 2.32% Coverage

IQ;	are there any obstacles?

RR;	Aaaa the challenges is that you find that ambulance is not working, mother delays at home to come to the facility, while she was told that she is supposed to deliver at the facility, she has delayed even to go to dispensary, so you will find that she has reached a dangerous stage and make you run here and there to try save her life

IR;	mm

RR;	mmh so there are challenges

IR;	mm

RR;	mm

IQ;	by using your experience, what things can cause a mother to be referred, from a health centre which gives delivery operations to district hospital? What can cause a mother to be referred?

RR;	mmm maybe a doctor is not there

IR;	mm

RR;	those people who work in theatre is not there

IR;	mm

RR;	mm or there is no blood; because you cannot do operation without blood bank

IR;	mm

RR;	mmh I think I have no more experience but those are the main reasons, and others can be minor things

IR;	mmh

RR;	mm

IQ;	do you think the way system in now can affect in one way or another the efficiency of assistant medical officers?

RQ;	which system?

IQ;	how does the referral system affect assistant medical officers to provide services of delivering through operations?

RR;	aaaa how does the system affect for instance a mother has come in late stage and failed to manage her and she arrives there is worse situation?

IR;	mm

RR;	mm another thing maybe we have said that you find that there is no car, or has seen that I can’t manage this case and there is no car at the same time

IR;	mm

RR;	so that also troubles the doctor

IR;	mm

RR;	to make decision

IR;	mm

RR;	mm

Reference 5 - 0.79% Coverage

IQ;	is there a positive side of effects?

RQ;	mm?

IQ;	are there good results on how the referral system can facilitate or becomes a burden for an assistant medical doctors to do an operation?

RR;	yah like I have said there are communications

IR;	mm

RR;	the doctor gets communication early and prepares his team ready to do the work

IR;	mm

RR;	if there is a car; it goes to pick him up and comes to operate on the patient so that he doesn’t delay that patient. If there is blood of course he can perform that operation with confidence yah

Reference 6 - 1.91% Coverage

IQ;	ok, thank you. When you look on the way structure of the referral system is, do you think that roles of assistant medical officer have changed?

RR;	mmmhm they have not changed

IR;	mm

RR;	mmmh

IQ;	what do you mean?

RQ;	when you say that they have changed do you mean due to his roles that he was doing has changed or?

IQ;	it means that maybe in the past referrals were done because you have said that the one who does referral

RR;	mm

IQ;	doctor in charge

RR;	mm

IQ;	of that particular place

RR;	mm

IQ;	that means that the doctor in charge in the current situation the way I understood you is that Assistant medical officers

RR;	mm

IQ;	in those many health facility, you said that the doctor in charge, it he was not assistant medical officers do you think his roles in referral would change? Bearing in mind that the in charge is the one who gives referrals?

RQ;	if the referrals were not done by the in charge

IQ;	I am looking the decision maker

RR;	mm

IQ;	in making referral

RR;	referral enhee

IQ;	you have said its Assistant medical officers in many cases

RR;	anhaa I have said it’s the in charge

IR;	in charge

RR;	ee in charge

IQ;	now….

RR;	the in charge can be someone else who is not Assistant medical officers

RR;	enhee

RR;	he could be a CO or even a nurse

RQ;	even a nurse?

RR;	yah

IQ;	Aaa she can be a nurse?

Reference 7 - 1.67% Coverage

RR;	yaah, other facilities have nurse only, and the in charge is a nurse and no doctor

IR;	Anhaa

RR;	mm

IQ;	now when you look at referral on the operation case

RR;	mm

IQ;	Do you think that roles of assistant medical officers changes due to referrals? I mean that as a decision maker, because on referrals we look at the person who feels in the forms

RR;	mm

IQ;	we look at that decision maker; he decides that this patient needs to be referred because there are two things…..aren’t there?

RR;	mm

IR;	there ….

RR;	the one who makes decision that this patient needs to be referred [mixed voices people talking] yah

IQ;	enhee, who makes decision for referral cases?

RR;	doctor

IQ;	doctor?

RR;	facility in charge

IR;	mmh

RR;	is the one who refers, for instance if the person in the labour ward fails to handle the case she will call the doctor on duty

IR;	mmh

RR;	that doctor is the one who will decide to refer that patient to another place

IR;	mm

RR;	he will write that referral case

IQ;	also it’s the doctor on duty?

RR;	doctor on duty

IQ;	and isn’t a must that he should…..

RR;	it’s not necessarily the head of the facility

Reference 8 - 1.43% Coverage

IQ;	I mean he won’t have his own decision, he should inform another person? What are those things?

RQ;	in the administration or?

IR;	any thing

RR;	of course he is supposed to inform others. What is he supposed to do is to inform others about those cases and they way he handles them. Cases that he faces and complications so other are informed that there are such things or a patient has delayed, and where was she delayed, I mean he should inform others about the whole system so that they know what is going on. Even if he is operating on a patient they should know what is he operating on? And why is he operating on her?

IR;	mm

RR; what is the problem in our area?	What is the big problem? So he should inform others about those things, and during decision making, he should inform others as well; that I have failed to handle this case and I want to refer it to a certain place, and tell them why he is referring that case- he must tell his colleagues that.

IR;	mm

RR;	yah

<Internals\\MK4000_W_06> - § 2 references coded [11.67% Coverage]

Reference 1 - 9.70% Coverage

IQ: How is your referral system here especially to the patient who needs cesarean section?

RR: If a patient comes for the first time, most of them go straight to the labour ward and get assessed by the labour ward nurse. If they notice any complication which they cannot manage they call the first doctor on call most of the times are clinical officers and he also asses the patient again and if he finds any problem he cannot manage, he calls the second on call who is the medical doctor or assistant medical officer. If it’s a case they can manage they take it to the theatre but if it’s a complicated case they refer it to a higher facility (St. Francis) with a written document and using an ambulance.

IQ: After referring how is the procedure of getting the feedback?

RR: Honestly there is no feedback maybe if the doctor is so concerned about the patient can make a phone call to ask for the wellbeing of the patient but there is no feedback in written form. Sometimes patients come to say thank you to the doctors or the referral hospital might call to give feedback when death occurs for the sake of internal auditing.

IQ: What challenges do you get in the whole process of referral?

RR: The main challenge is the transport system, when the car breaks down it can take two to three months to get repaired. Patients get hard time and here in Ifakara there are no taxes so they have to use Bajaj together with the escorting nurse, you can imagine the trouble they get or sometimes an escorting nurse have to take a motorcycle.

IQ: How does the referral system affects the performance of assistant medical officer especially on cesarean section?

RR: for example there is an emergency at night and the car is not there to go peek up the doctor, you might find the doctor is willing to come but there no transport so that is one of the challenges we are facing. Sometimes you remain puzzled when the doctor is around but the patient needs to be referred due to complications and there is no transport

Reference 2 - 1.97% Coverage

IQ: What is the responsibility of an assistant medical officer in the whole process of referral? Have the responsibilities changed according to time or they are still the same?

RR: At first the assistant medical officer were at the top and had final say on referral but now they cannot do that, they have to consult or call a medical doctor for approval. I think those are the few changes which have occurred.

<Internals\\MK4000_WF_02> - § 1 reference coded [2.47% Coverage]

Reference 1 - 2.47% Coverage

RR: Teachers are enough, however we are many, and we are so many compared with our teachers

IQ: You are from jobs, if you look at this training, for instance if you project at the time you will finish school do you think if this training will add something to you? On your confidence compared with the previous time before you join this training?

RR5: Because, we have worked previously and now we have gone to class again. I see that I have changed a lot, quite different from the previous time

IQ: Now in which area do you think you have changed?

RR5: We have changed in all areas, for instance on how to talk with a patient, how to treat her and things like that

RR3: I see that I have changed a lot compared to the time when I came. Before I came I was a CO, and on the side of OBS and GYN we were...as CO we were referring cases to higher level hospitals. But now I will not be referring anymore. When I get a patient I am supposed to act, when I fail to handle that case I will consult my senior, something different from when I was a CO. I was managing some, doing resuscitation and referring patients. So now I have changed. I can treat a patient, until when I see that I have failed to do so, and then I will find someone to help me

<Internals\\MK5000_T_03> - § 1 reference coded [3.14% Coverage]

Reference 1 - 3.14% Coverage

RR: The first thing that allows an AMO to perform his or her work is, his or her education. That is, he or she needs to have a medical diploma and that is what makes him or her assistant medical doctor. That is the first factor. The second factor is employment if this individual is employed at a hospital it directly makes him / her a medical doctor and there is also improvement, that is from time to time there are trainings and short courses to improve their knowledge and skills for instance maybe there is a new course in switching of treatment medicines, those HIV medicines, you know the ARVs, they get training on the changes like you know in the villages is where they are mostly involved and this is one of the sectors in which they have advanced themselves in this HIVs sector, so they get training, they gain skills, they gain knowledge required in doing their job. So those factors are the ones I think allow them to continue doing their work. The other thing I can speak on are these normal factors, the motivations they get at work, their recognition as medical sector employees that is also something that contributes to their functioning because if someone works and you don’t acknowledge him / her it really discourages them from continuing with work.

**DOMAIN: REFFERAL SYSTEM RECOMMENDATIONS**

<Internals\\MK1000_K_01> - § 1 reference coded [8.08% Coverage]

Reference 1 - 8.08% Coverage

IP: What do you recommend in order to improve the referral system?

RR: The government should ensure that every health facility in this country has an ambulance. I am sure it can, we see every Member of Parliament is given a very expensive luxurious car, how about the health facilities that saves life of people. They should cut down the money taken to luxurious and help saving lives of their voters.

IQ: Thank you very much, we have started to approach the end, but before coming to the last section let us now discuss about the skill mix in performance of caesarean section. Please tell us how the availability of other cadres involved in cesarean section tasks influence the performance of CS by AMOs

RR: Mmmh…you are really well prepared, I like you and your way…you ask questions that directly touch our day to day issues here. You know performing surgery is teamwork; you need an assistant surgeon, you need anesthetic person and a nurse and people from laboratory. So you need at least four people for a caesarean section. But here we have a serious shortage; sometimes we even use people from laboratory as assistant surgeons or circulating nurse. I am the only surgeon and have only one anaesthetist and she is my assistant surgeon.

IP: How do you explain the ability of other cadres you are working with in performing caesarean section?

RR: Their capacity is doubtful…it is very low, imagine my anaesthetist is a medical attendant who received only four months training in anesthesia and she is my everything here. If she is not there, no operation is carried out. For the nurses it is very worse they are all attendants.

IP: With that regards in the available health workers how do you reach decisions about caesarean section?

RR: Our anaesthetist is also our midwifery; she is experienced in deliveries. Most of the time we discuss together with other workers available on what we should do to this pregnant woman and finally we reach a decision. In short it is a team work decision.

<Internals\\MK1000_W_08> - § 1 reference coded [2.22% Coverage]

Reference 1 - 2.22% Coverage

IP; so how can the referral system be improved?

RR; (silent…..) hmhm…… hmhmmmm….I think first of all the government should fit in the issue of fuel for the ambulances in the general budget that they should set aside the budget that should cover for the fuel used at least in the whole year whenever there is an obstacle in transporting patients. So that we may be able to take them directly to the referral center and whenever the patient requires service that is not available in that particular facility let’s say Bugando then we should be able to refer them directly especially when it comes to staff members rather than being forced to refer them to the regional hospital where they will go through another doctor who will review them and grant a go ahead for the referral to the applicable facility. For example; you may have a cardiac patient, you know well that the patient requires an ECHO and that there is no an ECHO machine at the regional hospital but you have to refer the patient to the regional hospital so that a go ahead maybe granted to the applicable facility.

<Internals\\MK1000_W_09 (1)> - § 1 reference coded [2.04% Coverage]

Reference 1 - 2.04% Coverage

IQ: what is your opinion, on how we can improve our referral system?

RR: Infrastructure should be improved

IP: Infrastructure on referral?

RR: Yes. For instance you give referral, while the roads are bad, so a mother delays to arrive. So it becomes a problem.

IR: Hahahahahah okay. Anything else?

RR: Anything else?

RR: Those who receive referrals (a door is opening……). Before referring a person, you are supposed to make sure that the reasons for referring her are valid, [satisfy yourself]. IR:Mmmmmhu

<Internals\\MK2000_W_01> - § 1 reference coded [1.28% Coverage]

Reference 1 - 1.28% Coverage

RQ: how it can be improved?

IR: yes

RR: first, “…..” I haven’t understood…..improved how

IR: improvement of the whole system, for instance when you receive information of a patient (mmmmhh) when they come, getting information from where he / she is coming from (mmmh) what do you think if done would be much better.

RR: Mainly I think is communication, (mmmh) that is I can say if there was communication between where the patient is sent to and where he / she is from, (mmh) it could be improved much more and follow up could be made more attentively.

IR: okay

RR: Maybe if there was a person responsible who would follow up on these, who would follow up on where the patient is going, (yes) or who lets say would deal with all referrals, (yes) that would be a nice thing.

IR: okay

<Internals\\MK2000_W_05> - § 1 reference coded [1.13% Coverage]

Reference 1 - 1.13% Coverage

IP: How can this system be improved?

RR: This referral system? Yes I think the government should add on more budgets for reproductive health in general. Because these challenges of the patients to stay for a long time at the facilities is caused by low understanding. Again the problem of transport there are some facilities which are far away from the district hospital. You will find that the ambulance has problems or has gone somewhere else; because ambulances do other work as well

<Internals\\MK2000_W_07> - § 1 reference coded [3.94% Coverage]

Reference 1 - 3.94% Coverage

IQ: Thanks, what are your opinion in improving the referral system that is being used for now for example putting the challenges into consideration what else do you think should be done to improve it

RR: I don’t see if there is lot of improvements, maybe they should follow up on patients after arrival if operation was done, and if she was pregnant did she get a live baby so they should follow up if patient received management which was referred for.

IP: Is there special approach to that follow up or its individual based you just decide to know about your patient?

RR: There is no such follow up here, but I suggest it to be there even asking through phone using medical in charge of here and Ndanda

<Internals\\MK3000_W_01> - § 2 references coded [2.06% Coverage]

Reference 1 - 1.21% Coverage

RR: that is the first thing. The second thing is during referral an escort should be there

IR: ok

RR: so this nurse who is escorting a patient, and the drive that brings the patient here, want to be paid, while there is no ability to pay them in time

IR: Yes

RR: OC comes after several months, and when it comes you find that money is not there, although you have indicated in the budget

IR: ok

RR: this is failing now, and a person is discouraged. Two days ago, I took two patients today I have taken one person, and I have never been paid. So you tell him that it will come, so he will be doing his work just because it’s his responsibility, what should he do?

Iron

RR: that is the third area, the first one is fuel, second is ability to maintain those cars, and lastly incentives for those who escort patients

Reference 2 - 0.84% Coverage

RR: A motorcycle was brought here for referring patients, however it is not working well, it doesn’t turn right, where can you find roads that turn left only? None.

IR: None

RR: We have dismantled those motocycles and sent them to remote areas, because we need them to bring report to us before seventh of every month, so that we enter them in to DHS2. So we have given those motorcycles to peripheral areas and they use them to bring reports and not to refer patients.

IR: Ok, its right, thank you very much. Let’s talk about the infrastructure and equipment

RR: Mmhuu

<Internals\\MK3000_W_03> - § 1 reference coded [2.89% Coverage]

Reference 1 - 2.89% Coverage

IQ: Therefore…..what do you suggest to be done in the referral system so as to improve on? For more benefits?

RR: I would suggest for instance a mother comes from the village to a health centre

RR: the provider there

IR: mmhu

RR: should see if she can serve her at that moment she arrives

IR: mmhu

RR: She serves her, but if she thinks that she will fail to do so, then she should refer her early

IR: mmhu

RR: Rather than keeping her for two days, and later she sees that time has gone and bring her here

IR: Mmhu

RR: So you find that many mothers when they are brought here the babies die in their womb. So you operate on her and remove the baby to save the mother from death

IR: Mmhu

RR: and the source is that they have delayed her there for a long time

IR: Mmhu,

<Internals\\MK3000_W_07> - § 1 reference coded [1.22% Coverage]

Reference 1 - 1.22% Coverage

IQ: In your opinion, how can we solve or improve the challenges of the referral system?

RR: As I have said previously, if other stations could have theatres to perform scissor we could not have referrals from very far distances, for example health centers at Mkata and Kabuku don’t have theatres so all patients are referred here

<Internals\\MK3000_W_08> - § 1 reference coded [4.65% Coverage]

Reference 1 - 4.65% Coverage

IQ; ok and how is that referral system maybe the referral system can improve if we had opportunity to improve that whole referral process either to improve in giving or receiving?

RR; Eee the main thing there to improve first because the main thing that affects this maybe we go back in the beginning maybe is the financial status of the patient because someone tells you even when you send me there because there she will not be given treatment free of charge she must pay so one tell you even the fee to pay I don’t have and majority for instance if you take the other case your patient cant deriver in the normal way she must get caesarean section but even with that because of the problem she has she need to see a specialist maybe the heart specialist because she has this heart problem because of the state he/she is will tell you okay will agree you tell him/her but you need to go earlier you find other the pregnancy is still young not that but she must be seen by the physician maybe she will agree u tell her okay go and prepare yourself when you are ready come I will write a letter to go there okay they will answer you that they will come but you find maybe they left and go to witch doctor and what not because there is that she has been witched meaning these person cannot have this problem and this one now contributes a lot for this they believe more on witch doctors than hahaa but so apart from that we had to choose a doctor that will make put the cooperation with those witch doctors so that when a certain issue we ask him/her now to go and to discuss with them and telling them people eee when a person of this and this kind came here bring her fast to the hospital but even with that still there is the problem so you find if you tell her this she doesn’t believe it is a problem that she has she will tell you why I have given birth one time and at home there wasn’t a this problem and that thing makes the condition to continue being hard so she say she doesn’t have the money to go there but she has the one for the witch doctor and she will go there and treated locally anyhow you might come and find her again in the delivering stage and the labour and have already given birth now you we wrote you a letter to refer you and you didn’t have money so that also contribute to much extent in that referral systems so you find a person come have already started labour you start thinking so this one with this condition will we arrive Tanga! We won’t reach so we have to cancel we will do whatever we can here but depend on the patient condition there is this and this

<Internals\\MK4000_K_01 (2)> - § 1 reference coded [0.58% Coverage]

Reference 1 - 0.58% Coverage

IQ: in your opinion how do you think the referral system can be improved?

RR: infrastructures should be improved, during the winter season we use 12 to 16 hours to Ifakara, our roads are not good at all.

<Internals\\MK4000_K_03> - § 1 reference coded [1.98% Coverage]

Reference 1 - 1.98% Coverage

RR: What are your suggestions in improving the present referral system?

IQ: The main issue in the referral system is transport, it’s easier if a patient has to be referred and there is a reliable transport. But most of the times the patients’ car is not functioning, so we face transport difficulties.

<Internals\\MK4000_M_01> - § 1 reference coded [1.92% Coverage]

Reference 1 - 1.92% Coverage

1Q: Ok how can we improve this system?

RR: First of all feedback is very important. When somebody referred to the next level feedback should go to the next level whether positive or negative. Because the issue of finance is a problem since the nurse who serve that service need to be paid extra duty payment other need night allowances but there is a financial deficit. But even the night allowances are not paid on time.

IR: Mm

IR: Even that is a challenge. Another challenge is transport if possible every health center could have an ambulance bust of them don’t have ambulance even hospitals. But this is a challenge since referral is connected to transport.

<Internals\\MK4000_W_01> - § 1 reference coded [1.51% Coverage]

Reference 1 - 1.51% Coverage

IQ: How can referral system be improved?

RR: To improve the system there should be these PPM they should be allocated that it should be repaired within three months it is unfortunate that the ambulance are out. It is too expensive to repair them and for the referral system they should have good infrastructure for car and ambulance they should be in good position to work. The system has improved the caesarean section as for Mlimba which have few emergencies.

<Internals\\MK4000_W_02> - § 1 reference coded [1.06% Coverage]

Reference 1 - 1.06% Coverage

IQ; how can we improve the referral system?

RR; the facilities should be adequate, documentation and diagnosing aren’t the issues, the people are ready but the problem is that whenever we face any obstacles the staffs who are supposed to be nearby the hospital have no accommodations, so it happens that a patient fails to be attended by an AMO, that fails completely, so there should be more facilities but that is a challenge that can be solved, long term plan but for short term the ambulance should be working all the time and that is the major challenge that we see and on our side we should care tenderly for our patients

<Internals\\MK4000_W_03> - § 1 reference coded [2.64% Coverage]

Reference 1 - 2.64% Coverage

IQ;	we are finishing up, but what opinion do you have on improving referral system?

RR;	opinion! My opinion on our side

IQ;	anywhere

RQ;	anywhere?

IR;	Yes

RR;	opinion that I have is related to where we refer, there is a problem there

IQ ; which opinions do you have, even if you can’t go and tell them direcltly, they will receive your message in one way or the other

RR;	they should listen to us; they should listen to our problem, because it’s not that we want to refer those cases. No. Another challenge is about money at St Francis. Patients are hurt when we tell them that we are referring them to St. Francis. They become unhappy because of that challenge [money]. So that challenge is about money and reception. Reception should be good there

<Internals\\MK4000_W_04> - § 1 reference coded [1.56% Coverage]

Reference 1 - 1.56% Coverage

IQ;	and the way the referral system is now, do you think if it needs to be improved, what will be improved? Do you think there is anything to be improved in that system?

RR;	yah the system can be improved I don’t know how can I put it- maybe it can be improved by giving education to those mothers so that they are able to know those indicators early and go to hospital early

IR;	mm

RR;	mm I mean instead of waiting until she is referred, she should have referred herself early another thing is to have maternity waiting home, those mothers who live far can come early and wait near that facility waiting for operations or services

IR;	mmh

RR;	mm if they build maternity waiting home, when mothers are told that they have dangerous indications, they go early there waiting for the time to come so that they undergo cesarean sections

IR;	mmh

RR;	mm

IQ;	how about health facility level? How do you think that the referrals can be improved?

RQ;	in the health facility?

IR;	mm

RR;	to make sure that ambulances are working and fuel is there and should work effectively

<Internals\\MK4000_W_06> - § 1 reference coded [3.50% Coverage]

Reference 1 - 3.50% Coverage

IQ: What areas could you wish to be improved in the referral system?

RR: Transport is the first thing; transport should always be available and there should be an alternative if it’s not functioning. The second thing is feedback, there should be feedback of our patients we refer to know there wellbeing.

IQ: On the issue of feedback, is there a problem on the receiving hospitals or others?

RR: Receiving hospitals

IQ: Is there no procedure of asking for feedback?

RR: You can’t because in the referral form there is a portion for feedback (to be explained what was done there) which can be cutoff and sent back but they never do that. So maybe the referral hospitals should be emphasized on giving back the feedback.
