# Supplementary material for: Understanding barriers to implementing referral procedures in the rural and semi-urban district hospitals in Tanzania: Experiences of healthcare providers working in maternity units
Source: PLoS One. 2021 Aug 26;16(8):e0255475. doi: 10.1371/journal.pone.0255475 (PMC8389460; doi:10.1371/journal.pone.0255475)
Supplement: S1 Data — (ZIP) [file pone.0255475.s001.zip › Supporting documents/Interview Guide.doc]

**Appendix 1: INTERVIEW GUIDE: REFERRAL SYSTEM**

**Introduction**:

*Good morning/ good afternoon. My name is……….and this is……. (If two people are present). I/We am/are from Muhimbili University of Health and Allied Sciences. Thank you for giving me/us your time. We are here to learn about the ‘rational’ re-distribution of tasks among health workforce teams. Specifically, we are focusing on the implementation of referral system. We are not aiming at evaluating or critiquing the referral system or performance of caesarean section by the AMOs rather, to understand the establishment of this cadre in performing caesarean section to the contemporary situation.*

PART A: PARTICIPANTS’ DEMOGRAPHIC DATA

| **Basic Participant Information** | |
| --- | --- |
| Time of the beginning of interview: | Age: |
| Date of interview: | Sex: |
| Region: | Highest level of Education: |
| District: | Education specialty: |
| Professional Title: | Interviewer name: |
| Duration at work place: | Time of finishing the interview: |
| Duration in current position: | Participant Code: |

**PART B: EXPERIENCE WITH REFERRAL SYSTEM**

*Now that we have discussed the regulations in caesarean section tasks by AMOs, I would like to discuss with you on how the referral system influences the provision of adequate caesarean section*

1. How does the AMOs handle emergency obstetric referrals from the lower facilities? (Probe: Availability of transport and communication, availability of senior experts for caesarean section at referral level, referral protocols)
2. What are the available referral support systems for emergency obstetric services? (Probe: Vehicles, fuel, extra duty payment of drivers and health worker, maintenance)
3. How does the referral system influence the scale up of caesarean section tasks in your region? (Probe: Barriers, facilitators, challenges)
4. What are the lessons learnt if any regarding the referral system with regard to caesarean section tasks by AMOs in your district region?
5. In your own words, what else would you like to share with me regarding the referral system/s in his healthcare facility?

***Thank you very much for your time***
