# Supplementary material for: Understanding barriers to implementing referral procedures in the rural and semi-urban district hospitals in Tanzania: Experiences of healthcare providers working in maternity units
Source: PLoS One. 2021 Aug 26;16(8):e0255475. doi: 10.1371/journal.pone.0255475 (PMC8389460; doi:10.1371/journal.pone.0255475)
Supplement: Attachment — Submitted filename: Comments from Editor and Reviewers - Tanzania MC referral study.docx [file pone.0255475.s002.docx]

**Review of the Barriers to referral study for maternity care in Tanzania**

Note from Staff Editor Beryne Odeny (bodeny@plos.org): PLOS ONE considers qualitative and mixed-methods studies for publication. We recommend that authors use the COREQ checklist, or other relevant checklists listed by the Equator Network, such as the SRQR, to ensure complete reporting (http://journals.plos.org/plosone/s/submission-guidelines#loc-qualitative-research). In general, we would expect qualitative studies to include the following**: 1) defined objectives or research questions; 2) description of the sampling strategy, including rationale for the recruitment method, participant inclusion/exclusion criteria and the number of participants recruited; 3) detailed reporting of the data collection procedures; 4) data analysis procedures described in sufficient detail to enable replication; 5) a discussion of potential sources of bias; and 6) a discussion of limitations.**

**Comments from Academic editor – Charu C Garg**

**Background:**

Lines 92- talks about 2 delays – but mentions only two in the bracket. Make it more explicit

Line 95 – state the key point of the MoH guideline that the audience must know with reference to the referral procedure that you talk about changing in the conclusion

Put you research in perspective, what you know in the field and what you would be studying additional through your research. Some studies are mentioned from Tanzania, may need to elaborate a little more about their findings about barrier and also what do you know from international literature.

**Material and methods**

Material and methods:

The facilities chosen have bias towards rural district hospitals. Normally the types of facilities and where they are based make a difference. Need to clarify by increasing your sample with different types of facilities, or make your study conclusions for specific types of facilities and specific regions. Would also be useful to know the differences in these regions in terms of the health system readiness.

Was there a difference in rural and urban referrals – would help to bring it out. However, you only chose one semi urban facility. It would be useful to increase your sample especially in urban areas to bring out the implementation barriers in urban areas, if that is something that you think will be useful

Overall based on interviews of 12 personnel from 4 regions and specific facilities, may not be enough to make general conclusions about the regulatory policy for all types of facilities. The sample size need to be more structured and increased or make the conclusion for characteristics of specific region and facilities used in your study

While your methodology mentions the qualitative research method you are using is in depth interview and open ended surveys, also useful to specify type of qualitative research method you are using – narrative method or phenomenological or grounded theory etc. . Can you explain how coses were assigned and used for the data. The thematic analysis you have used is a good approach to your research.

Data analysis procedures should be described in sufficient detail to enable replication.

1. Data analysis has been done selectively – It Should be done for every question from all participants for the questions asked. You have nicely picked put the themes and subthemes, summarize what the participants felt about it.

Discussion and Conclusion refers only to the barrier in regulation and unavailability of transport in the abstract, but you also had the third them on communication which should be mentioned. It might be useful to provide examples from other countries how some of the barriers have a solution eg, transport barriers have been taken care of in India through a public private partnership

**Results**

The whole section on Participant’s characteristics is missing. Are the participants - those referring or those receiving the referral or both? Please clarify Theme 3 would be better understood with those receiving the referral.

Not clear how table 2 are findings. Seem more like the themes you would have put in your questionnaire to study. Please put the questionnaire as supplementary material. Also the columns should be reversed with sub-themes coming after themes. These themes would have come from your literature review and could have started to appear in introduction, but definitely take to material and methods.

In theme 1(ii) – What are the barriers in protocol as understood by the health worker

In theme 2 – what about the availability of staff for the ambulance?

Theme 3: Is discharge form not given for feedback about women’s condition?

All comments should be tabulated under a sub-theme to make it easier to follow. Also, for each subtheme it is important to know How many responses were received from a given type of facility. Based on just one comment from one provider we cannot make a conclusion that referral protocol has a problem. Further what is the problem and solution perceived by the providers and authors. Eg. for line 203 - could the solution be Grading the condition of the patient by MO and referring based on that. Or line 223-225: off duty emergencies to be allowed by junior staff. Later the MO should be able to assess and sign. Also availability of ambulance at night.

Lines 211-215 need to be more clearly written.

230-233 – Are those the part of referral guidelines.

265-267 – could ambulance be called from higher level facilities. There are some good examples of public private partnerships for ambulance services from India. May be useful to discuss in discussion.

**Discussion**

A lot of the beginning discussion could be in introduction. Discussion should follow from results. – Identifying the problems for each theme and providing a solution with support from the literature.

**Other comments:**

1. References need to be completely reformatted. Most references do not follow the PLOS One style.

PLOS uses the reference style outlined by the International Committee of Medical Journal Editors (ICMJE), also referred to as the “Vancouver” style. Example formats are listed below. Additional examples are in the [ICMJE sample references](http://www.nlm.nih.gov/bsd/uniform_requirements.html). Need to give first 6 authors et. al.

Example for published papers: Hou WR, Hou YL, Wu GF, Song Y, Su XL, Sun B, et al. cDNA, genomic sequence cloning and overexpression of ribosomal protein gene L9 (rpL9) of the giant panda (Ailuropoda melanoleuca). Genet Mol Res. 2011;10: 1576-1588.

Putting the references in the text also need to be corrected eg. [5,6] and not [5], [6] …several other places as well . Please check the PLOS one manuscript writing guide.

Reference 31 not mentioned in the text. Need to be checked. Why is ther a . before reference 9 and 10.

2. Several grammatical and spelling errors

Line 53, 87, 227 (lent), 327 (where not were),

472-476, 481-482 – Grammar not clear.

and many more

**Comments from Reviewer 1**

I reviewed a manuscript titled " Understanding barriers to implementing referral procedures in the rural and semi-urban district hospitals in Tanzania: Experiences from healthcare providers working in maternity units.

The manuscript is well written

The authors need to correct a few grammatical errors in the manuscript (e.g line 148, 149)

Methodology: The type of sampling strategy used was not clear. The authors stated the definition of convenience sampling on line 129, but did not clearly indicate if this was the strategy used in this study.

References: Please remove the period (.) in the beginning of reference 4,5,9 and 10

**Comments from Reviewer 2:**

The study is relevant in the country context and provides policy recommendations to address high MMR in Tanzania and other countries with similar kind of health system challenges. However, the language of the article could have been more reader friendly. I strongly suggest authors to work with a writing editor to address the grammatical errors, improve the flow and readability of the text.      

The authors may consider providing more information regarding “where” mothers are dying in the study area, means whether they are dying at home at the facilities, or in transit. Generally, the data related to place of maternal deaths are available in the MDSR (Maternal Death Surveillance and Response) reports. If Tanzania has MDSR system in place, the data could be analyzed to find out the weakest link in the referral system and findings would complement the qualitative findings of the study making it more statistically relevant.  

Authors have tried to refer and divide their findings as per the standard “Three Delays" model, however, it could have been more explicit in a simple and standard language.

Authors may please go through an article from Haiti with similar findings, link is given below:

Barnes-Josiah D, Myntti C, Augustin A. The "three delays" as a framework for examining maternal mortality in Haiti. Soc Sci Med. 1998 Apr;46(8):981-93. doi: 10.1016/s0277-9536(97)10018-1. PMID: 9579750.

**Reviewer 3:**

This is an important issue to be raised, timely referrals are important to save lives. This research  is based on a very small sample size (only 12 interviews) and the sample is also opportunistic. would suggest the  authors to have a bigger sample to be able to draw conclusions to bring a policy change on referrals.
the authors should do an English language check.
